# Distinct Frontoparietal Brain Dynamics Underlying the Co-Occurrence of Autism and ADHD

**DOI:** 10.1523/ENEURO.0146-23.2023

**Published:** 2023-07-11

**Authors:** Daichi Watanabe, Takamitsu Watanabe

**Affiliations:** 1 College of Letters and Science, University of California, Berkeley, Berkeley, CA 94720; 2 International Research Center for Neurointelligence, The University of Tokyo Institutes for Advanced Study, Tokyo 113-0033, Japan

**Keywords:** ADHD, autism, comorbidity, energy landscape analysis, intrinsic neural timescale, MRI

## Abstract

Previous diagnostic systems precluded the co-existence of autism spectrum disorder (ASD) and attention-deficit/hyperactivity disorder (ADHD) in one person; but, after many clinical reports, the diagnostic criteria were updated to allow their co-occurrence. Despite such a clinical change, the neurobiological bases underpinning the comorbidity remain poorly understood, and whether the ASD+ADHD condition is a simple overlap of the two disorders is unknown. Here, to answer this question, we compared the brain dynamics of high-functioning ASD+ADHD children with age-/sex-/IQ-matched pure ASD, pure ADHD, and typically developing (TD) children. Regarding autistic traits, the socio-communicational symptom of the ASD+ADHD children was explained by the same overstable brain dynamics as seen in pure ASD. In contrast, their ADHD-like traits were grounded on a unique neural mechanism that was unseen in pure ADHD: the core symptoms of pure ADHD were associated with the overly flexible whole-brain dynamics that were triggered by the unstable activity of the dorsal-attention network and the left parietal cortex; by contrast, the ADHD-like cognitive instability of the ASD+ADHD condition was correlated with the atypically frequent neural transition along a specific brain state pathway, which was induced by the atypically unstable activity of the frontoparietal control network and the left prefrontal cortex. These observations need to be validated in future studies using more direct and comprehensive behavioral indices, but the current findings suggest that the ASD+ADHD comorbidity is not a mere overlap of the two disorders. Particularly, its ADHD-like traits could represent a unique condition that would need a specific diagnosis and bespoke treatments.

## Significance Statement

Children with autism spectrum disorder (ASD) have cognitive rigidity and tend to persist in specific thoughts, whereas those with attention-deficit/hyperactivity disorder (ADHD) exhibit overly flexible cognition and have trouble with concentration. Despite such contrast, clinically, the two neurodevelopmental disorders often co-exist in one person. How can such a co-occurrence happen? By investigating the global and local brain dynamics, this study found that the comorbidity of ASD and ADHD is not a simple overlap of the two conditions. Instead, the cognitive instability seen in ASD+ADHD children was underpinned by unique brain dynamics that were not observed in pure ADHD. These findings indicate that the comorbid condition would need a bespoke diagnosis and treatment.

## Introduction

Relations between autism spectrum disorder (ASD) and attention-deficit/hyperactivity disorder (ADHD) have not been simple. ASD has cognitive rigidity as one of its core symptoms (Lopez et al., 2005; Uddin et al., 2015; Watanabe et al., 2019a; Uddin, 2021), whereas ADHD tends to show overly flexible and unstable cognition (Kuntsi and Klein, 2011; Das et al., 2014). Previous diagnostic systems, such as DSM-IV-TR and ICD-10, precluded their co-existence in one person (Gargaro et al., 2011). However, >15% of ADHD children were found to exhibit ASD traits (Kotte et al., 2013; Cooper et al., 2014), and >40% of ASD children were reported to possess ADHD characteristics (Kaat et al., 2013; Salazar et al., 2015; Joshi et al., 2017; Rong et al., 2021). Largely because of such clinical studies, the diagnostic systems were updated and now allow the co-occurrence of these two prevalent neurodevelopmental disorders (Young et al., 2020; American Psychiatric Association, 2022).

In contrast to this major change in clinical practice, the behavioral property of the ASD+ADHD comorbidity is still under debate (Berenguer et al., 2018; Benallie et al., 2021), and its biological mechanisms are not fully understood (Lau-Zhu et al., 2019).

In behavioral studies, the cognitive properties of ASD+ADHD individuals (Bühler et al., 2011; Mayes et al., 2012; Craig et al., 2015, 2016; Dajani et al., 2016; Colombi and Ghaziuddin, 2017; Yerys et al., 2019a) have been continually examined, and some of them suggest that ASD+ADHD cohorts would have unique executive functions; but multiple systematic reviews concluded the behavioral observations on the comorbidity were incongruent with each other (Berenguer et al., 2018; Benallie et al., 2021).

Biological knowledge of the brain mechanisms underpinning the ASD+ADHD condition is also limited. The neurobiological bases of pure ASD (Bourgeron, 2015; Watanabe and Rees, 2017; Demetriou et al., 2018; Hong et al., 2019; Watanabe et al., 2019b; Roy and Uddin, 2021) and pure ADHD (Castellanos et al., 2002; Dickstein et al., 2006; Rubia et al., 2010; Castellanos and Proal, 2012; Cortese et al., 2012; Hart et al., 2012, 2013; Cai et al., 2018; de Lacy and Calhoun, 2018; Kaboodvand et al., 2020; Jeong et al., 2021; Shappell et al., 2021; Rajagopal et al., 2022; Yin et al., 2022) have been intensively studied, and their similarities/distinctiveness were also identified in genes (Ronald et al., 2008; Rommelse et al., 2011; Cross-Disorder Group of the Psychiatric Genomics Consortium et al., 2013; Martin et al., 2014; Satterstrom et al., 2019) and brain architectures (Christakou et al., 2013; Lim et al., 2013; Chantiluke et al., 2014; Aoki et al., 2017; Dajani et al., 2019; Lake et al., 2019; Lau-Zhu et al., 2019; Yerys et al., 2019b; Boedhoe et al., 2020; Ohta et al., 2020; Sáenz et al., 2020; Harikumar et al., 2021; Curtin et al., 2022; Hansen et al., 2022; Hoogman et al., 2022; Safar et al., 2022). In contrast, except for five MRI studies (Solomon et al., 2009; Chantiluke et al., 2014; Nickel et al., 2018; Dajani et al., 2019; Yerys et al., 2019b), no human neurobiological research reported brain mechanisms of ASD+ADHD comorbidity. In addition, two of the five exceptional works did not conduct direct comparisons between ASD+ADHD, pure ASD and pure ADHD (Solomon et al., 2009; Yerys et al., 2019b). One of the other three studies identified disorder-general/-specific neural signatures for atypical temporal foresight but did not investigate neural architectures for any core symptom of either disorder (Chantiluke et al., 2014). The other two works reported no atypical neural representation that correlated with any symptom of ASD or ADHD (Nickel et al., 2018; Dajani et al., 2019). Consequently, it remains unknown whether the comorbid condition is a simple overlap of pure ASD and pure ADHD or represents a distinct neurodevelopmental disorder (Berenguer et al., 2018; Lau-Zhu et al., 2019; Hours et al., 2022).

Partly because of such behaviorally and biologically limited knowledge, some clinicians cast doubt on the concept of the ASD+ADHD comorbidity itself and warned that some standard medications for ADHD symptoms, such as the administration of amphetamine stimulants, might cause undesirable effects on ASD+ADHD individuals (Hours et al., 2022).

Here, to address this situation, we aimed to identify the neural mechanisms underpinning the ASD+ADHD condition by directly comparing the global and local brain dynamics of high-functioning ASD+ADHD children with age-/sex-/IQ-matched pure ASD, pure ADHD and typically developing (TD) individuals (Table 1).

**Table 1:** Demographic data

	ADHD200 (NYU)			ABIDE (NYU)					ADHD vsASD+ADHD	TD in ADHD200vs TD in ABIDE
	ADHD	TD	ADHD vs TD	ASD+ADHD	ASD	TD	ASD+ADHDvs ASD	ASD+ADHD/ASD vs TD		
*N*	30	29		33	30	38				
Age	8.8 ± 0.9(7–11)	9.0 ± 1.4(7–13)	*p *=* *0.2	8.1 ± 1.9(5–13)	8.5 ± 2.3(5–13)	8.7 ± 1.4(5–11)	*p *=* *0.5	*p *>* *0.2	*p *=* *0.1	*p *=* *0.4
Sex	7 females	10 females	*p *=* *0.1	3 females	4 females	6 females	*p *=* *0.6	*p *>* *0.2	*p *=* *0.1	*p *=* *0.1
FIQ	109.1 ± 14.2	111.4 ± 12.8	*p *=* *0.4	111.8 ± 17.8	109.4 ± 14.0	111.4 ± 11.1	*p *=* *0.6	*p *>* *0.2	*p *=* *0.8	*p *=* *0.6
VIQ	109.5 ± 12.7	113.3 ± 14.5	*p *=* *0.2	112.0 ± 16.6	107.5 ± 14.9	111.7 ± 13.6	*p *=* *0.3	*p *>* *0.2	*p *=* *0.9	*p *=* *0.6
PIQ	105.9 ± 15.4	106.9 ± 12.9	*p *=* *0.8	112.2 ± 20.2	109.2 ± 17.3	108.7 ± 12.3	*p *=* *0.5	*p *>* *0.4	*p *=* *0.2	*p *=* *0.9
CPRS ADHD Index	73.2 ± 8.1	44.5 ± 5.6	*p *<* *10^–5^	—	—	—	—	—	—	—
CPRS ADHD inattention	71.5 ± 9.0	44.1 ± 4.6	*p *<* *10^–5^	—	—	—	—	—	—	—
CPRS ADHD hyperactivity	70.3 ± 11.5	46.8 ± 7.2	*p *<* *10^–5^	—	—	—	—	—	—	—
SRS total raw score	—	—		91 ± 30.6	92.5 ± 30.6	20.3 ± 11.4	*p *=* *0.8	*p *<* *10^–5^	—	—
ADIR social	—	—		17.1 ± 6.9	19.1 ± 5.3	—	*p *=* *0.2	—	—	—
ADIR verbal	—	—		15.0 ± 5.3	16.0 ± 4.2	—	*p *=* *0.4	—	—	—
ADIR RRB				5.2 ± 2.6	6.5 ± 4.2		*p *=* *0.03			
ADIR Total	—	—		37.2 ± 12.0	41.5 ± 10.1	—	*p *=* *0.2	—	—	—

Technically, we first applied the energy landscape analysis (Watanabe et al., 2014; Ezaki et al., 2017; Kang et al., 2017; Watanabe and Rees, 2017) to resting-state functional MRI (rsfMRI) datasets and depicted global brain state dynamics for each participant group. Then, we identified which aspect of the brain state dynamics was specific to each symptom. Next, we performed exploratory whole-brain analyses of the intrinsic neural timescales (Hasson et al., 2008, 2015; Honey et al., 2012; J.D. Murray et al., 2014; Baldassano et al., 2017; Watanabe et al., 2019b; Wolff et al., 2022) and narrowed down a focal neural area whose atypically unstable neural activity triggered the atypical global brain dynamics. We then integrated these findings using a mediation analysis and illustrated the unique brain mechanisms underpinning the ASD+ADHD comorbidity. The robustness of these results was confirmed with two independent rsfMRI datasets. In the final part, we conducted a behavioral experiment to examine the validity of the assumption on the metrics for ADHD-like traits of ASD+ADHD individuals.

## Materials and Methods

### Datasets

This case-control study analyzed rsfMRI data that were recorded from the following four types of cohort: children with ADHD only, those with ASD only, those with ASD and ADHD (ASD+ADHD), and TD controls. The datasets of the pure ADHD and corresponding TD children were shared on a website for the ADHD200 project (ADHD-200 Consortium, 2012), whereas those of the pure ASD, ASD+ADHD and corresponding TD children were hosted on a website for the ABIDE project (Di Martino et al., 2014, 2017). We chose these datasets (1) because all the rsfMRI data were recorded at the same single site (New York University) by essentially the same research team with almost the same scan parameters and (2) also because their sample size was the largest among such single-site datasets. The other single-site datasets with smaller sample sizes were used in the confirmatory test. All the data were collected with the approval of the ethics committees or institutional review committees of the recording institutes (ADHD-200 Consortium, 2012; Di Martino et al., 2014, 2017). Note that, for strict comparison, we did not merge the two TD groups into one in both the main analysis and confirmatory test.

### Diagnosis and demographic data

We selected participants based on their age (5 < age < 13), IQ (80 ≤ full/verbal/performance IQ ≤ 140), and head motion during echoplanar imaging (EPI) data recording (mean head motion ≤ 3 mm). We set the age range to reduce the effects of adolescence. The IQ scores of the pure ADHD and corresponding TD children were quantified by the *Wechsler Intelligence Scale for Children*, Edition 4. The IQ scores of the pure ASD, ASD+ADHD and corresponding TD children were measured with the Wechsler Abbreviated Scale of Intelligence. The mean head motion was calculated in the preprocessing procedures of EPI data as described below.

ASD was diagnosed based on Autism Diagnostic Interview-Revised (ADI-R) and DSM-IV-TR. ADHD was diagnosed by clinical experts in accordance with Conners’ Parent Rating Scale-Revised (CPRS), long version.

For the pure ASD group, we selected ASD children in the ABIDE dataset who met all the above conditions and had no comorbidity.

For the pure ADHD group, we chose ADHD children with no other comorbidity in the ADHD200 dataset. We employed all the three types of ADHD (hyperactive, inattentive, and combined) to ensure a sufficient sample size.

For the ASD+ADHD group, we selected autistic children with ADHD from the participants enrolled in the ABIDE dataset. In the ADHD200 data, we found no ADHD children who showed ASD symptoms and met the above criteria for participant selection. In the ABIDE dataset, the comorbid ADHD in the ASD children was determined when the ASD children met all the requirements for ADHD except for criterion E in the DSM-IV-TR, which excluded the co-occurrence of ADHD in ASD individuals. In other words, we selected children who would be given ASD+ADHD dual diagnosis in the DSM-5.

For the TD groups, we selected age-/sex-/IQ-matched TD children from the ADHD200 and ABIDE datasets, respectively.

As a result, this study used the data recorded from 30 high-functioning pure ADHD children, 29 TD children for the pure ADHD group (TD in ADHD200), 33 high-functioning ASD+ADHD children, 30 high-functioning pure ASD children, and 38 TD children for the ASD+ADHD and pure ASD children (TD in ABIDE; Table 1).

### Symptom metrics

For both the pure ASD and ASD+ADHD groups, the severity of ASD symptoms was evaluated with the ADI-R scores that were shared in the ABIDE datasets. We did not use the scores of Autism Diagnostic Observation Schedule (ADOS), which were also provided in the ABIDE dataset, to evaluate autistic traits. The range of the ADI-R scoring system is sufficiently broad to evaluate the details of ASD symptoms; but the ADOS has narrower score ranges and, in particular, can give only a limited range of scores for cognitive rigidity (i.e., RRB). Given this, we used the ADI-R scores to assess the ASD symptoms.

The severity of ADHD symptoms seen in the pure ADHD children was assessed based on the CPRS scores (CPRS ADHD Index, CPRS Inattention score and CPRS hyperactivity score) that were provided in the ADHD200 dataset.

The severity of ADHD-like traits of the ASD+ADHD group was not quantified with the CPRS scores since, as stated above, all the ASD+ADHD participants were selected from the ABIDE dataset, which did not contain the CPRS scores. Instead, here, we inferred such ADHD-like behaviors of the ASD+ADHD children based on the subscore for the repetitive restricted behaviors (RRB) in Autism Diagnostic Interview-Revised (ADI-R); that is, this study assumed that ASD+ADHD children with lower RRB scores should have more unstable/flexible cognition and manifest ADHD-like behaviors.

This inference is primarily grounded on previous literature showing that (1) the symptoms of high-functioning ADHD individuals, in particular, their hyperactivity, are correlated with their cognitive flexibility/instability (Semrud-Clikeman et al., 2010; Das et al., 2014) and (2) the cognitive instability measured by psychological experiments is inversely associated with the RRB score assessed in clinical tests for ASD symptoms (Lopez et al., 2005; Watanabe et al., 2019a; Cissne et al., 2022). We can find face validation for this assumption in other prior findings that (1) pure ADHD children had more flexible and unstable cognition compared with TD (Kuntsi and Klein, 2011) and pure ASD individuals (Ozonoff and Jensen, 1999; Sergeant et al., 2002; Corbett et al., 2009; Lawson et al., 2015) and (2) the RRB score was likely to be lower in pure ADHD individuals than in pure ASD children (Brierley et al., 2021; Mellahn et al., 2022). In fact, claim (2) was confirmed even in the current dataset, which showed that the ASD+ADHD children had lower RRB scores than the pure ASD group (*t*_(61)_ = 2.1, *p *=* *0.03 in a two-sample *t* test; Table 1).

In addition, we conducted a behavior experiment to provide supportive evidence for this rationale (see below, Behavioral experiment).

### MRI data acquisition

All the MRI data were recorded in a 3T scanner (MAGNETOM Allegra, Siemens) at New York University while the participants were asked to be relaxed with their eyes open. Functional MRI data were obtained using an echoplanar imaging (EPI) sequence (for ADHD200 and ABIDE I: TR 2 s, TE 15 ms, 33 slices, interleaved, flip angle 90°, spatial resolution 3.0 × 3.0 × 4.0 mm, scan time 6 min; for ABIDE II: TR 2 s, TE 30 ms, 34 slices, interleaved, flip angle 82°, spatial resolution 3.0 × 3.0 × 3.0 mm, scan time 6 min). Anatomical images were recorded with a T1-weighted sequence (TR 2.53 s, TE 3.25 ms, flip angle 7°, spatial resolution 1.3 × 1.0 × 1.3 mm).

### Preprocessing of rsfMRI data

We preprocessed the EPI data with SPM12 (https://www.fil.ion.ucl.ac.uk/spm/software/spm12/) in the same manner as in our previous studies applying the energy landscape analysis or intrinsic neural timescale analysis to rsfMRI data (Watanabe and Rees, 2017; Watanabe et al., 2019b). First, we discarded the first five images and conducted realignment, unwarping, slice timing correction, normalization to the standard template (ICBM 152) and spatial smoothing (Gaussian kernel with 8 mm of full-width at half maximum). We then removed the effects of head motion, white matter signals, cerebrospinal fluid signals and global signals before performing bandpass temporal filtering (0.01–0.1 Hz). We found no significant difference in any of the six parameters for the head motion between the groups (*p *>* *0.4).

### Overview of energy landscape analysis

**Figure 1.**
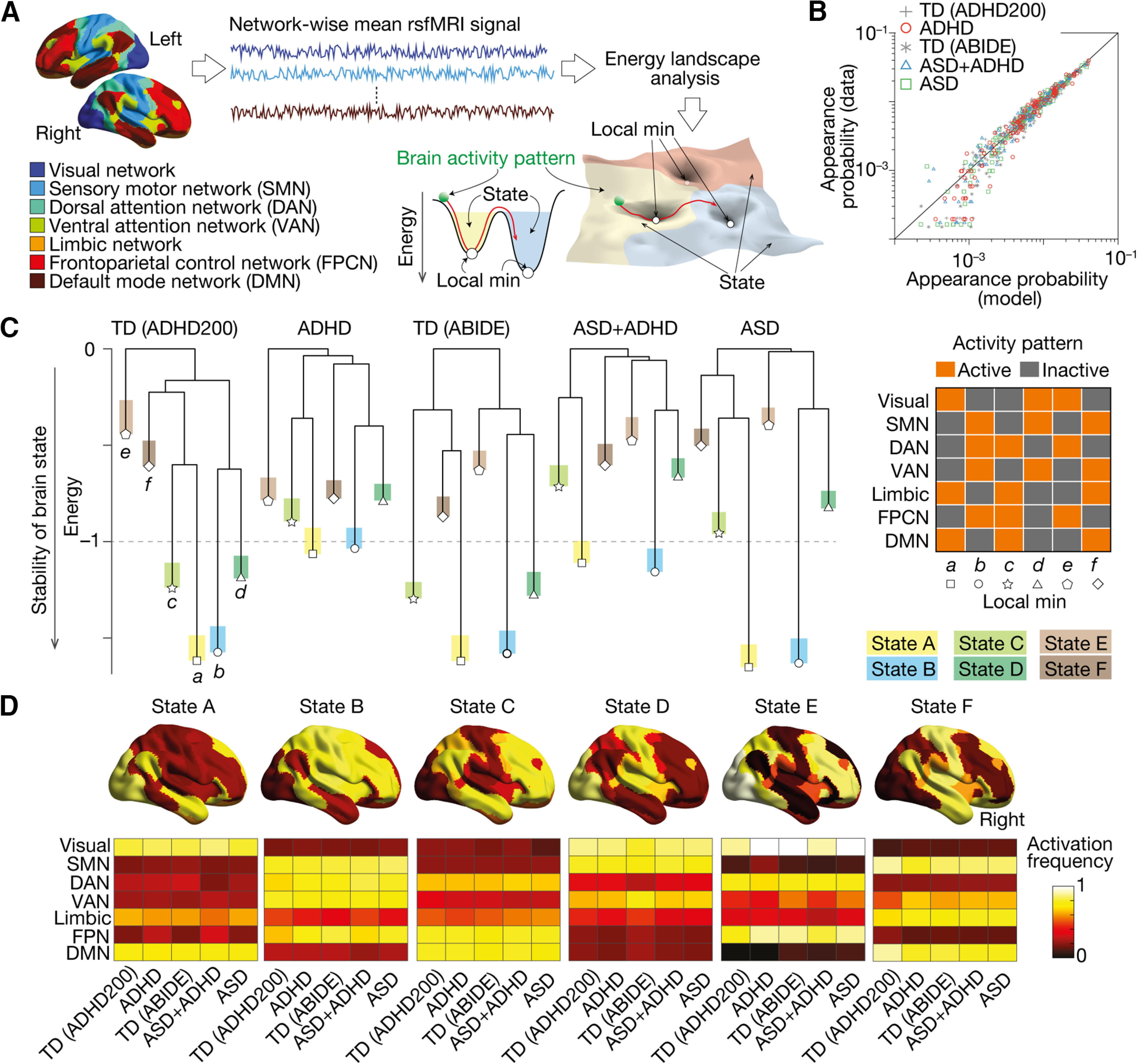
Six brain states determining global neural dynamics. ***A***, We performed energy landscape analysis to examine the global neural dynamics. After parcellating the brain into seven functionally distinct networks, we fitted a pairwise maximum entropy model (MEM) to the network-wise rsfMRI signals and identified the structure of the energy landscape for each group. In the energy landscape, local minima represented the most stable brain activity patterns in their neighboring areas, and basins (attractors) indicate the sets of the brain activity patterns that can be summarized into the corresponding local minimum. ***B***, The pairwise MEM was accurately fitted to the rsfMRI data in all the participant groups (>97.5%). ***C***, The dendrograms, so-called disconnectivity graphs, show the structures of the energy landscapes. All the participant groups shared the same six local minima (local min *a*–*f*), whose activity patterns were displayed in the right panel. ***D***, The six brain states (States A–F) corresponding to the six local minima were similar between the five participant groups (*r *>* *0.91). TD, typically developing. ADHD, attention-deficit/hyperactivity disorder. ASD, autism spectrum disorder. ASD+ADHD, a comorbid condition of ASD and ADHD. TD (ADHD200), TD data stored in the ADHD200 project. TD (ABIDE), TD data stored in ABIDE project.

The energy landscape analysis conducted here was essentially the same as that in our previous studies (Watanabe et al., 2014; Watanabe and Rees, 2017; Watanabe, 2021). Here, to investigate network-based brain state dynamics, we adopted a widely used brain parcellation system (Yeo et al., 2011; Hansen et al., 2022; Saggar et al., 2022) that divides the cerebral cortex into the following seven functionally distinct networks (Fig. 1*A*): visual network, sensory-motor network (SMN), dorsal attention network (DAN), ventral attention network (VAN), limbic network, frontoparietal control network (FPCN), and default mode network (DMN). We adopted this brain segmentation system mainly because our previous study using basically the same parcellation system succeeded in capturing ASD-specific brain dynamics. In addition, the amount of the current dataset is not enough for an accurate energy landscape analysis adopting a smaller ROI definition: the energy landscape analysis with *N* nodes requires data with 2*^N^*^–1^ timepoints for sufficiently accurate estimation; therefore, given the sample size of the current datasets, we could not adopt brain parcellation systems consisting of smaller regions of interest (ROIs).

Technically, we first calculated the mean rsfMRI signal for each network at each time point in each participant. We then binarized the network activities using the whole-brain average fMRI signal as a threshold. This binarization procedure balanced the numbers of active and inactive states and should improve the accuracy of the following analysis (Watanabe et al., 2013). We repeated this for every time point and obtained seven binary time-series data for each participant.

Using this dataset, we then conducted the energy landscape analysis. In short, first, we concatenated the binary data across participants in the same group and fitted a pairwise maximum entropy model (MEM) to them with a gradient ascent algorithm. We used the concatenated data so that we could achieve high accuracy of model fitting: in fact, because of the short fMRI scanning time (here, 6 min), we could not fit the pairwise MEM to individual data with sufficient accuracy.

This MEM fitting allows us to infer a hypothetical energy value for every activity pattern. Based on this energy value, we then built so-called dysconnectivity graphs (Fig. 1*C*), which showed structures of the energy landscapes and clarified local minima and corresponding basins (i.e., attractors). To avoid the risk of arbitrary reverse inference (Poldrack, 2006), we did not put psychologically meaningful labels on the brain states. Finally, we investigated the brain state dynamics on the energy landscapes by a random-walk simulation in a Markov chain Monte Carlo method with the Metropolis–Hastings algorithm (Girvan and Newman, 2002; Massen and Doye, 2005). Using this random-walk simulation, we quantified brain state transition frequencies, which were validated by looking into the individual empirical rsfMRI data.

Theoretically, the group-level random-walk simulation is supposed to capture the brain state dynamics more accurately than the examination of the individual empirical rsfMRI data, because the numerical simulation can give a sufficiently long brain-state history for us to calculate all the types of brain-state transitions. In contrast, rare brain-state transitions are likely to be missed in the relatively short empirical rsfMRI data. Although, such an individual assessment of brain state dynamics based on each fMRI dataset is known to be somewhat correlated with the results of the random-walk simulation (Watanabe et al., 2014; Watanabe and Rees, 2017). Based on such differences and previous observations, we first conducted the group-level random-walk simulation to exploratorily find disorder-specific/common neural dynamics; then, aiming to link such neural dynamics to disorder severity, we quantified individual brain state dynamics by looking into each empirical fMRI time-series data.

### Energy landscape analysis: model fitting

To quantify network-based brain state dynamics, we adopted a brain parcellation system (Yeo et al., 2011; Hansen et al., 2022; Saggar et al., 2022) that divides the cerebral cortex into the seven functionally distinct networks (Fig. 1*A*). After calculating the mean rsfMRI signal for each network at each time point in each participant, we binarized the network activities using the whole-brain average fMRI signal as a threshold (+1 for active and –1 for inactive). We repeated this for every time point and obtained seven binary time-series data for each participant. An activity pattern of the seven networks at time point *t* was described such as $V^{t}=[\sigma_{1}^{t},\sigma_{2}^{t},...,\sigma_{N}^{t}]$, where $\sigma_{i}^{t}$ represents a binary activity of network *i* at time *t* (i.e., $\sigma_{i}^{t}=+1or\;-1$) and *N* denotes the number of the networks (here, *N *=* *7).

We then concatenated the binary data across participants in the same group and fitted a pairwise MEM to them. This simple model consisted of two parameters, $h_{i}$ and $J_{ij}$. The *h_i_* is supposed to show the basal activity of network *i*, whereas $J_{ij}$ should represent a pairwise interaction and coupling strength between network *i* and network *j*. The $h_{i}$ and $J_{ij}$ were determined so that the average of the model-based network activity ${〈{〈\sigma }_{i}〉〉}_{m}$ and the average of the model-based pairwise interactions ${〈{〈\sigma }_{i}\sigma_{j}〉〉}_{m}$ are sufficiently close to the average of the empirical network activity $〈\sigma_{i}〉$ and the average of the empirical pairwise interaction $〈\sigma_{i}\sigma_{j}〉$, respectively. The $〈{〈\sigma_{i}〉〉}_{m}$ was defined as $\Sigma_{\ell =1}^{2^{N}}\sigma_{i}\left(V_{\ell }\right)P\left(V_{\ell }\right)$ and the ${〈{〈\sigma }_{i}\sigma_{j}〉〉}_{m}$ was defined as $\Sigma_{\ell =1}^{2^{N}}\sigma_{i}\left(V_{\ell }\right)\sigma_{j}\left(V_{\ell }\right)P\left(V_{\ell }\right)$, where $\sigma_{i}\left(V_{k}\right)$ is the activity of network *i* in the activity pattern *V_k_* and $P(V_{k})$ is the appearance probability of the neural activity pattern $V_{k}$. The $P\left(V_{k}\right)$ is given as $e^{-E\left(V_{k}\right)}/\Sigma_{\ell =1}^{2^{N}}e^{-E(V_{\ell })}$, where $E\left(V_{k}\right)=-\Sigma_{i=1}^{N}h_{i}\sigma_{i}\left(V_{k}\right)-\left(1/2\right)\Sigma_{i=1}^{N}\Sigma_{j=1}^{N}J_{ij}\sigma_{i}\left(V_{k}\right)\sigma_{j}\left(V_{k}\right).$ Based on these definitions, we adjusted *h_i_* and *J_ij_* until the ${〈{〈\sigma }_{i}〉〉}_{m}$ and $〈{〈\sigma_{i}\sigma_{j}〉〉}_{m}$ were approximately equal to the $〈\sigma_{i}〉$ and $〈\sigma_{i}\sigma_{j}〉$ with a gradient ascent algorithm.

The accuracy of this model fitting was evaluated by calculating a proportion of Kullback–Leibler (KL) divergence in this second-order model (*D*_2_) to that in the first-order model (*D*_1_) as follows (Watanabe et al., 2013, 2014; Watanabe and Rees, 2017; Watanabe, 2021): (*D*_1_ – *D*_2_)/*D*_1_.

### Energy landscape analysis: hierarchy between local minima

Next, we examined the structure of each group-specific energy landscape, which was defined as a graph of brain activity patterns *V_k_* (*k *=* *1, 2, …, 2*^N^*). In the graph, two activity patterns were regarded as adjacent if and only if their difference was seen at only one network activity.

In the energy landscape, we first searched for local energy minima, whose energy values were smaller than those of all the *N* adjacent patterns. We then visualized and quantified the hierarchical structures between the local minima by building so-called disconnectivity graphs in the following procedures (Watanabe et al., 2014; Watanabe and Rees, 2017; Watanabe, 2021): (1) We prepared a hypercube graph, in which each node (i.e., each brain activity pattern) was adjacent to the *N* neighboring nodes. (2) We set a threshold energy level, *E*_threshold_, at the largest energy value among the 2*^N^* nodes. (3) We then removed the nodes whose energy values $E\left(V_{k}\right)$ were greater than *E*_threshold_. (4) We examined whether each pair of local minima was still connected by a path in the slightly disconnected graph. (5) We repeated steps (3) and (4) after changing *E*_threshold_ down to the next largest energy value. We stopped these procedures when all the remaining local minima were isolated. (6) Based on the obtained results, we built a hierarchical tree, disconnectivity graph, whose leaves (i.e., terminal nodes down in the tree) represented the local minima and internal nodes indicated the branching points of different local minima.

### Energy landscape analysis: classification to brain states

Using this disconnectivity graph, we then classified all the brain activity patterns into one of basins (attractors) with a corresponding local minimum at their bottoms. First, we picked up node *i* from the 2*^N^* nodes. If any of its neighbor nodes had a smaller energy value than the node *i*, we moved to such a node. Otherwise, we did not move and classified the node as a local minimum. We repeated this procedure until we reached any of the local minima. The initial node *i* was then assigned to the basin of the local minimum that we finally reached. This procedure was repeated for all the 2*^N^* nodes, which allowed us to classify all the brain activity patterns on the energy landscape, except for nodes on the saddles, into any of the basins. In this study, these basins were regarded as brain states (Fig. 1*A*).

### Energy landscape analysis: brain state dynamics

Finally, we probed the brain state dynamics on the energy landscapes by a random-walk simulation that was based on a Markov chain Monte Carlo method with the Metropolis-Hastings algorithm (Girvan and Newman, 2002; Massen and Doye, 2005). This simulation allowed a brain activity pattern *V_i_* to move only to a neighboring pattern *V_j_*. Technically, we first chose one of such neighboring patterns randomly and then determined whether actual movement to the pattern occurred or not at the probability $P_{ij}=min[1,e^{E\left(V_{i}\right)-E(V_{j})}]$. In other words, when *V_i_* was more unstable than *V_j_* [i.e., $E\left(V_{i}\right)>E(V_{j})$], the brain activity pattern always moved from *V_i_* to *V_j_*. In the meantime, this probability setting left some room for moving to *V_j_* even if *V_i_* was more stable than *V_j_* [i.e., $E\left(V_{i}\right)<E(V_{j})$], which prevented the brain activity pattern from being trapped in a local minimum forever.

For each group, we repeated this random walk 10^5^ steps with a randomly chosen initial pattern, which resulted in a trajectory of the brain activity pattern such as $\left[V^{1},V^{2},...,V^{10^{5}}\right]$. After discarding the first 100 steps to reduce the effects of the initial condition, we then classified all the $V^{t}$ into either of the brain states and converted $\left[V^{101},V^{102},...,V^{10^{5}}\right]$ to, for example, [State A, State C, State B, State D, …]. We then counted how long each brain state continued in the trajectory (dwelling time) and how often one brain state transited to another state (transition frequency).

These indices based on the group-level energy landscape analysis were validated by looking into the individual empirical rsfMRI data. For example, for each participant, we directly converted its binary time-series data into a brain state trajectory and counted the transition frequency between specific brain states.

### Intrinsic neural timescale analysis

To examine local neural dynamics, we applied voxel-wise intrinsic neural timescale analysis (Hasson et al., 2008, 2015; Honey et al., 2012; J.D. Murray et al., 2014; Baldassano et al., 2017; Watanabe et al., 2019b; Wolff et al., 2022) to the preprocessed rsfMRI data. Note that this analysis of intrinsic neural timescale analysis did not use the binarized data that were employed in the energy landscape analysis. First, we estimated an autocorrelation function (ACF) of the rsfMRI signal of each voxel (time bin = TR) and then calculated the sum of ACF values in the initial period where the ACF showed positive values. The upper limit of this period was set at the point where the ACF hits zero for the first time. We repeated this procedure for every voxel and applied spatial smoothing to the brain map (Gaussian kernel, full-width at half maximum = 8 mm), which improved the signal-to-noise ratio. This whole-brain map was used as an intrinsic timescale map.

We conducted this analysis on every participant in all the groups and compared the maps between the groups using a random-effects model. We searched for brain regions showing significant differences (*p*_FDR_ < 0.05; Watanabe et al., 2019b).

### Confirmatory tests

We examined the reproducibility of the main analyses using two independent datasets collected at Kennedy Krieger Institute (KKI) and Oregon Health and Science University (OHSU; Table 2). Basically, we conducted the same analyses as in the main study. In the intrinsic neural timescale analysis, we did not perform the whole-brain voxel-wise analysis but a region of interest (ROI) analysis: the ROIs were defined as 4-mm spheres around the same coordinates as those of the left SFG and IPS found in the main analysis.

**Table 2:** Demographic data for confirmatory tests

	ADHD200			ABIDE				
	ADHD	TD	ADHD vs TD	ASD+ADHD	ASD	TD	ASD+ADHD vs ASD	ASD+ADHD/ ASD vs TD
KKI dataset								
*N*	15	29		18	7	74		
Age	10.5 ± 1.7	10.1 ± 1.2	*p *=* *0.4	9.9 ± 1.3	9.0 ± 1.2	10.0 ± 1.1	*p *=* *0.1	*p *>* *0.1
Sex	6 females	13 females	*p *=* *0.8	4 females	2 females	27 females	*p *=* *0.7	*p *>* *0.2
FIQ	100.2 ± 12.8	105.5 ± 7.5	*p *=* *0.2	100.1 ± 12.1	106.4 ± 15.6	105.5 ± 6.4	*p *=* *0.4	*p *>* *0.1
VIQ	103.1 ± 14.9	108.4 ± 10.1	*p *=* *0.2	106.4 ± 15.2	112.8 ± 18.4	110.2 ± 11.0	*p *=* *0.6	*p *>* *0.4
PIQ	105.3 ± 14.3	106.1 ± 10.3	*p *=* *0.8	98.7 ± 10.6	110.3 ± 5.6	104.1 ± 9.4	*p *=* *0.1	*p *>* *0.1
CPRS ADHD Index	72.3 ± 10.3	45.1 ± 4.4	*p *<* *10^–5^	—	—	—	—	—
CPRS ADHD inattention	71.3 ± 10.9	45.3 ± 4.9	*p *<* *10^–5^	—	—	—	—	—
CPRS ADHD hyperactivity	71.6 ± 11.0	46.3 ± 4.7	*p *<* *10^–5^	—	—	—	—	—
ADIR social	—	—	—	21.1 ± 5.1	20.9 ± 6.9	—	*p *=* *0.9	—
ADIR verbal	—	—	—	14.3 ± 5.1	15.1 ± 4.5	—	*p *=* *0.9	—
ADIR RRB	—	—	—	5.1 ± 1.6	5.6 ± 1.5	—	*p *=* *0.5	—
OHSU dataset								
*N*	17	23		12	17	16		
Age	9.5 ± 1.0	9.4 ± 1.2	*p *=* *0.6	12.7 ± 1.5	11.6 ± 1.7	11.7 ± 1.2	*p *=* *0.1	*p *>* *0.1
Sex	5 females	11 females	*p *=* *0.2	1 female	0 female	2 females	*p *=* *0.2	*p *>* *0.1
FIQ	110.2 ± 13.4	116.8 ± 8.9	*p *=* *0.1	99 ± 20.1	106.4 ± 18.2	110.0 ± 8.8	*p *=* *0.3	*p *>* *0.1
CPRS ADHD inattention	72.9 ± 7.2	47.2 ± 7.0	*p *<* *10^–5^	—	—	—	—	—
CPRS ADHD hyperactivity	67.5 ± 14.3	45.3 ± 4.7	*p *<* *10^–5^	—	—	—	—	—
ADIR social	—	—	—	19.2 ± 6.6	19.8 ± 5.4	—	*p *=* *0.8	—
ADIR verbal	—	—	—	15.8 ± 4.8	16.6 ± 4.9	—	*p *=* *0.7	—
ADIR RRB	—	—	—	4.8 ± 2.9	8.2 ± 3.4	—	*p *=* *0.008	—

### Statistics and data availability

Effects of multiple comparisons were addressed with Bonferroni correction except for the cases of the whole-brain analysis of the intrinsic neural timescales, in which we adopted FDR correction (Watanabe et al., 2019b). All the MRI data used here are available on ADHD200 and ABIDE sites. Essentially the same code used for the current energy landscape analysis was shared in our previous work (Ezaki et al., 2017).

### Behavioral experiment

Because of the limited demographic information in the rsfMRI datasets used here, we assumed that the degree of the ADHD-like traits in the ASD+ADHD children should be related to cognitive overflexibility and could be inferred by their RRB scores in ADI-R. Albeit indirectly, we tested this assumption by conducting a behavioral experiment employing 30 TD adults (seven females, 22.7 ± 2.3 years old, FIQ/PIQ/VIQ = 113.8 ± 5.6/117.5 ± 9.3/109.8 ± 8.7, mean ± SD). This experiment was approved by an institutional ethics committee at The University of Tokyo, and all the participants provided written informed consents and were financially compensated for their participation.

First, all the participants completed two self-assessment-based questionnaires: autism spectrum quotient (AQ) for the evaluation of their autistic traits (Baron-Cohen et al., 2001; Wakabayashi et al., 2006) and Conners’ Adult ADHD Rating Scale–Self-Report: Long Version (CAARS) for the quantification of their ADHD traits (Conners et al., 1999). For each participant, we then calculated an RRB-related score by adding “Attention switching score” to “Local details” in the AQ. To evaluate the ADHD-like trait, we calculated the “ADHD hyperactivity score” based on the CAARS. We focused on the hyperactivity score since the aforementioned main analysis highlighted the link between ADHD-specific neural dynamics and its hyperactivity trait.

Second, they underwent a spontaneous task-switching test, which was designed to assess cognitive flexibility/rigidity both in TD and autistic individuals (Arrington and Logan, 2004; Poljac et al., 2012; Watanabe et al., 2019a).

In the spontaneous task-switching test, we presented the participants with a series of visual stimuli consisting of four marks with different shapes and brightness. For each visual stimulus, the participants were asked to conduct either a shape task or a brightness task: in the shape task, they were required to find the preinstructed shape (e.g., circle); in the brightness task, they were asked to identify the brightest figure. The participants were also asked to complete either of the two tasks as accurately and quickly as possible. When they did not respond even 3 s after the stimulus onset, the next stimulus set was presented automatically.

To make the choice of the task as random as possible, we asked the participants, “You have to choose which task to perform on each trial. Ideally, you should perform each task randomly but on about half of the trials. Sometimes you will repeat the same task and sometimes you will switch from one to another. We don’t want you to count the number of times you’ve done each task or alternate strictly between tasks to make it sure you do each one half the time. Just try to do them randomly.” This instruction was used in the previous studies employing this psychological paradigm (Arrington and Logan, 2004; Poljac et al., 2012; Watanabe et al., 2019a).

The participants practised the two tasks separately until they were able to respond correctly (≥95% of accuracy) and quickly (reaction time ≤ 2 s). Then, the participants underwent five 3-min runs of this test.

After this test, we retrospectively inferred which task was selected for each trial. The participants rarely responded to any stimuli in a way in which we could not make such an inference (the proportion of the unclassifiable trials ≤1.2%). After excluding the unclassifiable trials, we counted how long each participant repeated the same task (= task-repetition length).

Finally, we compared the associations between the RRB-related score in AQ, “ADHD hyperactivity” measured by CAARS-S (T-score), and the task-repetition length in the spontaneous task-switching test.

## Results

This study analyzed the rsfMRI data recorded from the high-functioning ASD+ADHD, pure ASD, pure ADHD and two groups of TD children (Table 1). In all the following analyses, no statistically significant difference was found between the two TD cohorts; but, for strict comparison, we did not merge the two TD groups and used them as independent controls.

### Six brain states defining global neural dynamics

We investigated the global brain state dynamics using energy landscape analysis (Watanabe et al., 2014; Ezaki et al., 2017; Kang et al., 2017; Watanabe and Rees, 2017), which enables us to depict the complex spatiotemporal changes of whole-brain neural activity as dwelling in and transitions between the parsimonious number of different brain states (Fig. 1*A*).

First, we confirmed that the pairwise maximum entropy model, a basis of the energy landscape analysis, was accurately fitted to the rsfMRI data in all the participant groups (fitting accuracy >97.5%; Fig. 1*B*), which supports the validity of the following analyses.

Then, we examined the structures of the energy landscapes (Fig. 1*C*). All the participant groups shared the same six local minima (local min *a*–*f*), and the six brain states corresponding to the six local minima (state A–F) were also similar between the groups (*r *>* *0.91; Fig. 1*D*).

However, the six brain states showed different depths and stability in different patient groups (Fig. 1*C*).

In the two TD groups, their six brain states were classified into three types: the two deepest, i.e., the most stable, brain states (states A and B), two relatively shallow states (states C and D), and two unstable states (states E and F).

In the pure ASD children, the gaps between the stable and unstable brain states were widened: the stable brain states (states A and B) were deeper than the corresponding TD children, whereas the other brain states (states C–F) were shallower compared with the control.

In contrast, the gaps were reduced in the pure ADHD children: the depths of the relatively stable states (states A–D) were decreased, and the unstable states (states E and F) were deepened.

As for the energy landscape structure of the ASD+ADHD children, it was basically a shallower version of that of the pure ASD group. Only their states E and F were slightly deeper than those of the pure ASD individuals.

### Brain state dynamics

**Figure 2.**
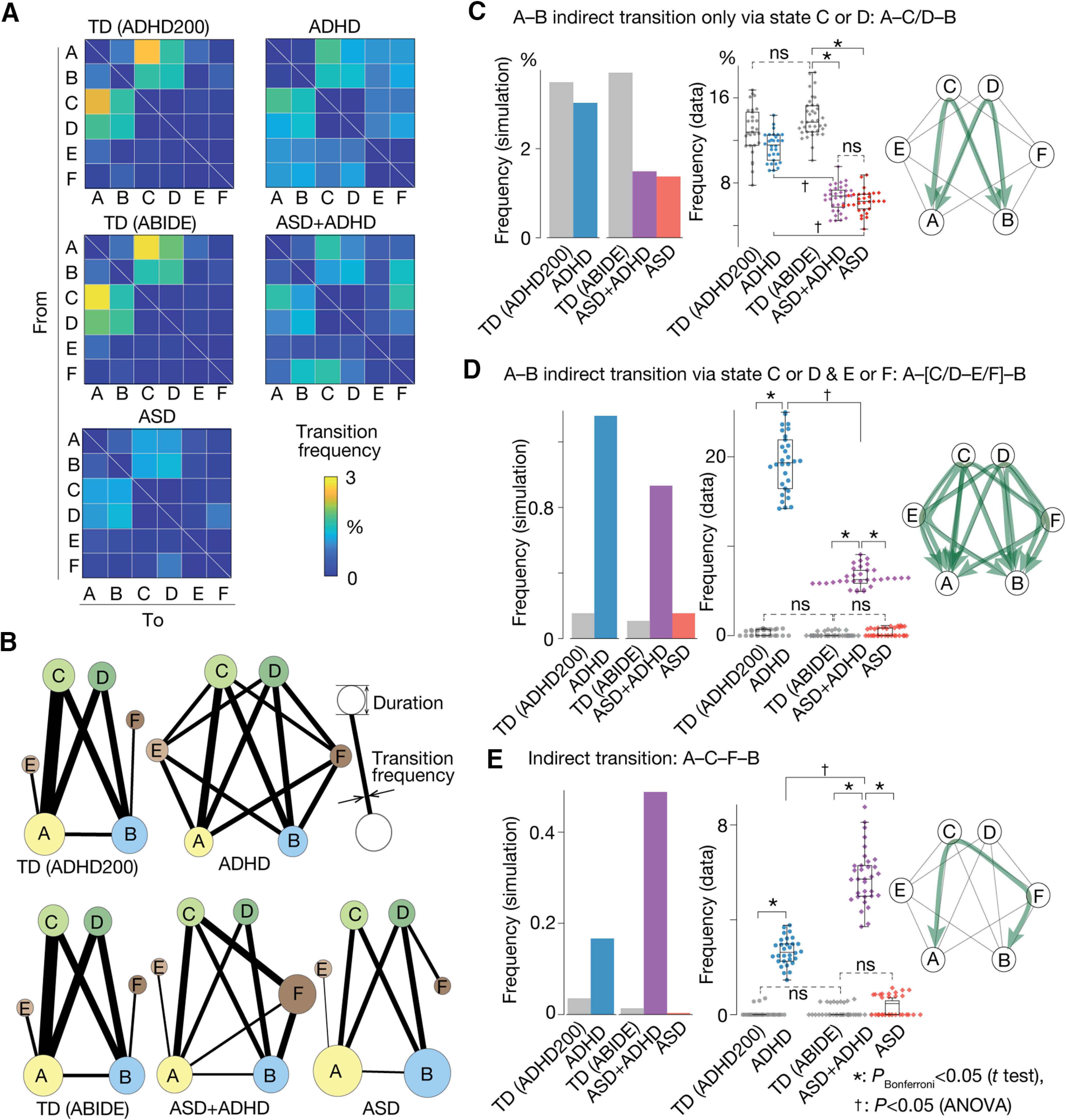
Brain state dynamics. ***A***, Transition frequency matrix. A cell (*i*, *j*) represents the frequency of the transition from brain state *i* to *j*, which was calculated by a random-walk simulation. ***B***, The graphs show the transition frequency (thickness of the lines) and duration (radius of the circle). ***C–E***, Three brain state transitions whose frequencies appeared to be specific to ASD/ADHD symptoms: panel ***C*** indicates the ASD symptom-specific reduction in the A–C/D–B transition frequency; panel ***D*** represents ADHD-specific enhancement of the A–[C/D–E/F]–B transition; panel ***E*** shows the ASD+ADHD comorbidity-specific increase in the A–C–F–B transition frequency. In every panel, the left bar graphs are based on the random-walk simulation, whereas the middle graphs are based on the empirical data. The right network schemata represent the patterns of the corresponding brain state transitions. *p_Bonferroni_ < 0.05 in a two-sample *t* test. ^†^*p *<* *0.05 for interaction in a two-way ANOVA.

Next, we examined how different brain dynamics were yielded on these structurally distinct energy landscapes. To this end, we conducted random-walk simulations on the energy surfaces and calculated the interstate transition frequencies and duration time of each state (Fig. 2*A*,*B*). Such simulation-based calculations were confirmed by counting how often these transitions actually happened in the empirical rsfMRI data.

In particular, we assessed the frequency of the so-called indirect transitions, which were defined as movements between the most stable brain states (states A and B) via the other states (states C, D, E or F). We focused on these transitions because our previous study successfully identified ASD-specific neural fingerprints in the atypically low frequency of such an indirect transition (Watanabe and Rees, 2017).

As a result, we found the following three indirect transitions whose frequencies showed atypical increases/decreases in symptom-specific manners.

First, the indirect transition via either state C or D (A–C/D–B; Fig. 2*C*, right panel) was atypically infrequent in the pure ASD and ASD+ADHD groups compared with the control (*t *>* *18.6, *p*_Bonferroni_ < 0.05, Cohen’s *d *>* *4.4 in two-sample *t* tests), whereas such a reduction was not seen in the pure ADHD children (*t*_(57)_ = 2.0, *p*_Bonferroni_ > 0.05; Fig. 2*C*).

Second, the indirect transition via either state C or D and either state E or F (A–[C/D–E/F]–B; Fig. 2*D*, right panel) was seen more often in the pure ADHD and ASD+ADHD groups than the TD children (*t *>* *31.5, *p*_Bonferroni_ < 0.05, *d *>* *8.2), while such an elevation was not found in the pure ASD group (*t*_(57)_ = 2.4, *p*_Bonferroni_ > 0.05). Moreover, the magnitude of this atypical increase in the A–[C/D–E/F]–B transition was significantly larger in the pure ADHD children than in the ASD+ADHD individuals (*F*_(1,126)_ = 457.5, *p *<* *10^−5^, $\eta^{2}$ = 0.16 in a two-way ANOVA; Fig. 2*D*).

Third, we found that the indirect transition between states A and B via states C and F (i.e., A–C–F–B transition; Fig. 2*E*, right panel) characterised the ASD+ADHD group. This indirect transition occurred more frequently in the pure ADHD and ASD+ADHD groups than in the other cohorts (*t *>* *26.2, *p*_Bonferroni_ < 0.05, *d *>* *6.8). In addition, this transition frequency was significantly higher in the ASD+ADHD children than in the pure ADHD individuals (*F*_(1,126)_ = 158.8, *p *<* *10^−5^, $\eta^{2}$ = 0.09; Fig. 2*E*).

Note that, for any of the three indirect transitions, no significantly different frequency was found between the two TD groups (*p *>* *0.7 in two-sample *t* tests).

### Brain state dynamics and symptoms

We then examined associations between these atypical brain dynamics and symptoms. Here, the autistic symptoms in the pure ASD and ASD+ADHD children were assessed with their ADI-R scores, and the symptoms of the pure ADHD individuals were quantified with their CPRS scores. Because of the limited data availability, the ADHD-like cognitive instability in the ASD+ADHD children was inferred based on their ADI-R RRB score; that is, we assumed that a lower RRB score indicated higher cognitive instability. For a more detailed justification of this assumption, please see Materials and Methods, Symptom metrics.

**Figure 3.**
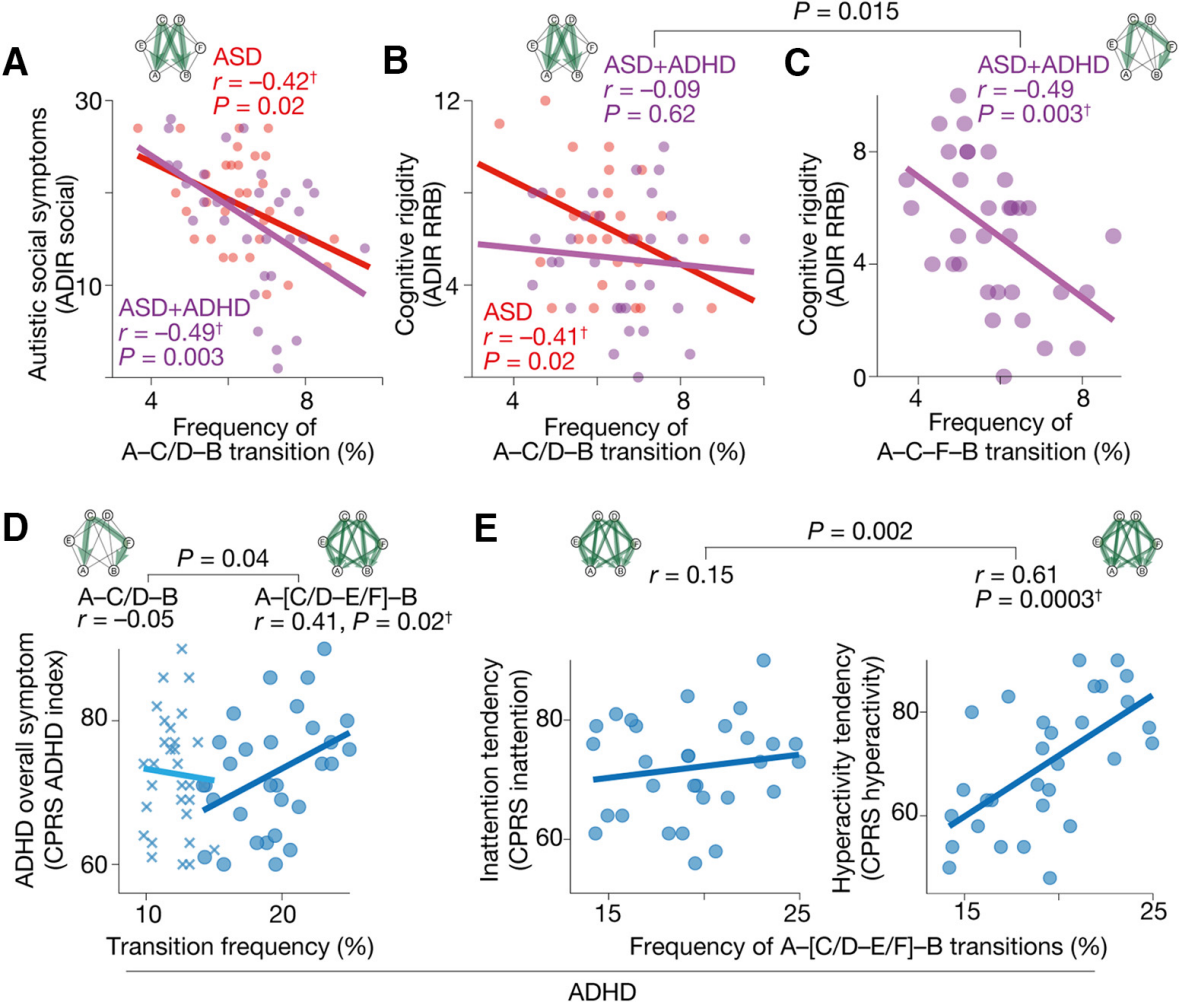
Brain state dynamics and symptoms. Three brain-state transitions exhibited symptom-specific atypical frequencies. ***A***, In both the pure ASD and ASD+ADHD groups, the severity of the autistic socio-communicational symptom (ADI-R social) was negatively correlated with the A–C/D–B transition frequency. ***B***, This A–C/D–B transition frequency explained the cognitive rigidity (ADI-R RRB) of the pure ASD children but did not that of the ASD+ADHD individuals. ***C***, Instead, the cognitive rigidity of the ASD+ADHD children was correlated with their atypically frequent A–C–F–B transition. ***D***, The ADHD symptom in the pure ADHD children was not explained by this A–C–F–B transition frequency but by the A–[C/D–E/F]–B transition frequency. ***E***, In particular, the atypical increase in the A–[C/D–E/F]–B transition frequency was specifically correlated with the hyperactivity tendency in the pure ADHD group. ^†^*p*_Bonferroni_ < 0.05.

In the pure ASD, the atypical decrease in the A–C/D–B transition frequency was linked with the severity of both the socio-communicational symptoms (*r* = –0.42, *p*_Bonferroni_ < 0.05; Fig. 3*A*) and the RRB symptom (*r* = –0.41, *p*_Bonferroni_ < 0.05; Fig. 3*B*), which is consistent with our previous work (Watanabe and Rees, 2017).

**Figure 4.**
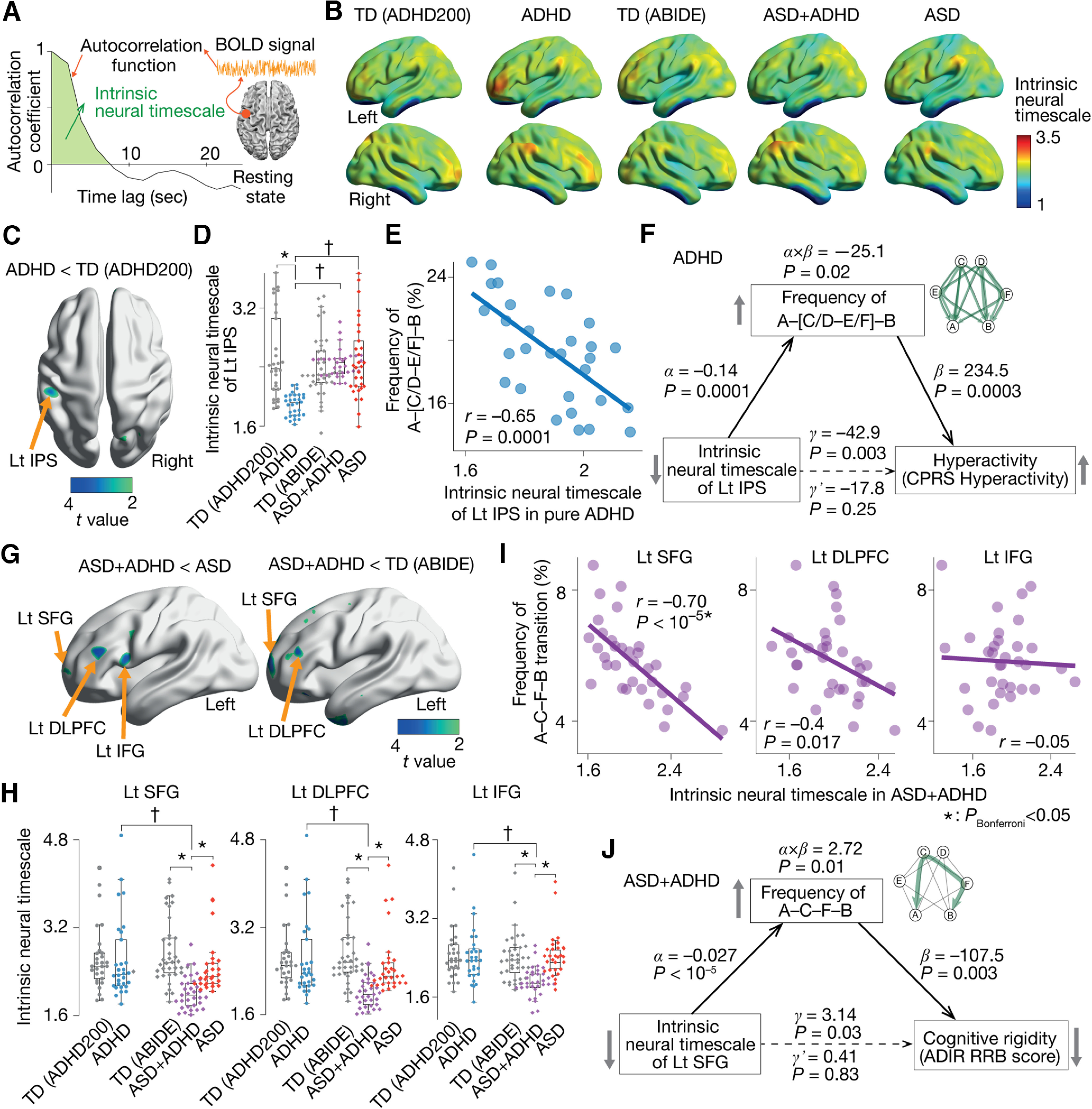
Local neural dynamics and global brain state dynamics. ***A***, To identify brain areas that induced atypical brain state dynamics in the pure ADHD and ASD+ADHD children, we examined the intrinsic neural timescales for all the brain regions. The neural timescale was defined as the area under the curve of the autocorrelation function. Brain areas with shorter neural timescales are thought to be sensitive to neural inputs and likely to exhibit an unstable and fluctuating neural signal. ***B***, For each of the participant groups, we obtained an average whole-brain map of the intrinsic neural timescale. ***C***, ***D***, In the pure ADHD children, only the left inferior parietal sulcus (IPS) showed a significantly shorter neural timescale compared with the TD individuals (*p*_FDR_ < 0.05; ***C***). The neural timescale of the brain area was also shorter than that of the pure ASD and ASD+ADHD groups (***D***). ***E***, This atypically shorter neural timescale in the left IPS in the pure ADHD children was correlated with their atypically frequent A–[C/D–E/F]–B transition. ***F***, A mediation analysis demonstrated that, in the pure ADHD individuals, their atypically shorter intrinsic neural timescale in the left IPS induced their frequent transition along the A–[C/D–E/F]–B pathway, which resulted in their hyperactive behavior. ***G***, ***H***, In the ASD+ADHD children, the neural timescales in the left superior frontal gyrus (SFG), dorsolateral prefrontal cortex (DLPFC), and inferior frontal gyrus (IFG) were significantly shorter than those of the pure ASD group (*p*_FDR_ < 0.05; ***G***), those of the pure ADHD children and those of the corresponding TD individuals (***H***). ***I***, Among the three regions, only the neural timescale of the left SFG showed a significant correlation with the atypical A–C–F–B transition frequency in the ASD+ADHD children. ***J***, A mediation analysis indicated that the short neural timescale of the left SFG induced the frequent A–C–F–B transition, which reduced the cognitive rigidity in the ASD+ADHD individuals. The significantly large values of the α, β, and γ validate our application of the mediation analysis to the current datasets. The statistical significance of the α×β (indirect effect) and the insignificance of the γ’ support our conclusions. ^†^*p*_Bonferroni_ < 0.05 in a two-way ANOVA. *p_Bonferroni_ < 0.05 in a two-sample *t* test.

In contrast, the autistic symptoms of the ASD+ADHD children were not fully explained by such atypically stable neural dynamics: their socio-communicational symptom was associated with the atypical reduction in the A–C/D–B transition frequency (*r* = –0.49, *p*_Bonferroni_ < 0.05; Fig. 3*A*), whereas their RRB symptom was not (*r* = –0.09, *p *=* *0.62; Fig. 3*B*). Instead, their RRB symptom was negatively correlated with their atypical increase in the A–C–F–B transition (*r* = –0.49, *p *=* *0.003; Fig. 3*C*). Given that a lower RRB score indicates more flexible cognition (Lopez et al., 2005; Watanabe et al., 2019a; Cissne et al., 2022) and such cognitive instability is related to ADHD symptoms (Semrud-Clikeman et al., 2010; Das et al., 2014), this result suggests that the ADHD-like trait in the ASD+ADHD children is linked with the atypically frequent A–C–F–B transition.

In the pure ADHD group, by contrast, their symptoms were not explained by this A–C–F–B transition frequency (*r* = –0.05, *p *=* *0.79); instead, the severity of their overall ADHD symptoms was correlated with their atypically enhanced A–[C/D–E/F]–B transition (*r *=* *0.41, *p *=* *0.02; Fig. 3*D*). In particular, the A–[C/D–E/F]–B transition frequency was correlated with their hyperactivity, specifically (*r *=* *0.61, *p *=* *0.0003; Fig. 3*E*). Note that, in the ASD+ADHD children, the frequency of the A–[C/D–E/F]–B transition did not account for their cognitive instability (*r* = –0.14, *p *=* *0.43).

Taken together, these findings indicate that the autistic social symptoms of high-functioning ASD+ADHD children are based on the same neural mechanisms as those of pure ASD individuals, whereas their ADHD-like cognitive instability is attributable to unique brain dynamics that are not related to any symptom of pure ADHD children.

### Brain state dynamics and intrinsic neural timescale

The above results highlight the double dissociation between the pure ADHD symptom and the ADHD-like traits in the ASD+ADHD comorbidity: the overly frequent A–[C/D–E/F]–B transition was associated with pure ADHD but not with the ADHD-like cognitive instability of the ASD+ADHD comorbidity, whereas the atypical enhancement of the A–C–F–B transition was linked with the ADHD-like behavior of the comorbid condition but not with pure ADHD.

To identify the origins of such double dissociation in the global brain dynamics, we examined the local neural dynamics of the pure ADHD and ASD+ADHD children. Technically, we performed intrinsic neural timescale analysis (Hasson et al., 2008, 2015; Honey et al., 2012; J.D. Murray et al., 2014; Chen et al., 2015; Baldassano et al., 2017; Runyan et al., 2017; Watanabe et al., 2019b; Wolff et al., 2022; Fig. 4*A*) for the entire brain in a voxel-wise manner (Fig. 4*B*) and searched for focal brain regions whose unstable neural activities were related to the atypical global brain state dynamics. In theory, brain areas with shorter neural timescales should yield fluctuating neural activities, destabilize the global brain dynamics, and induce more frequent brain-state transitions.

In the pure ADHD children, we identified the left inferior parietal sulcus (IPS; *x* = –50, *y* = –30, *z* = 42 in MNI coordinates) as a single region that showed a significantly shorter intrinsic neural timescale compared with the control group (*t*_(57)_ = 4.9, *p*_FDR_ < 0.05; Fig. 4*C*). This region’s neural timescale in the pure ADHD group was also shorter than in the ASD+ADHD individuals (*F*_(1,126)_ = 27.7, *p*_Bonferroni_ < 0.05, $\eta^{2}$ = 0.15) and pure ASD children (*F*_(1,126)_ = 15.5, *p*_Bonferroni_ < 0.05, $\eta^{2}$ = 0.1; Fig. 4*D*).

As expected, such an atypically short neural timescale in the left IPS was associated with the atypically higher frequency of the A–[C/D–E/F]–B transition (*r* = –0.65, *p *=* *0.0001; Fig. 4*E*). Moreover, mediation analysis indicated that this shortened neural timescale of the left IPS increased the A–[C/D–E/F]–B transition frequency, which resulted in the hyperactivity of the pure ADHD children (Fig. 4*F*).

In the ASD+ADHD children, the left superior frontal gyrus (SFG; *x* = –22, *y* = 62, *z* = 12), dorsolateral prefrontal cortex (DLPFC; *x* = –44, *y* = 32, *z* = 20) and inferior frontal gyrus (IFG; *x* = –50, *y* = 10, *z* = 14) had significantly shorter neural timescales than the pure ASD group (*t*_(61)_ > 4.1, *p*_FDR_ < 0.05; Fig. 4*G*, left). Particularly, the neural timescales of the left SFG and DLPFC in the ASD+ADHD children were shortened even compared with the TD individuals (*t*_(69)_ > 4.8*, p*_FDR_ < 0.05; Fig. 4*G*, right).

Among these three frontal regions, only the left SFG was significantly correlated with the A–C–F–B transition frequency (*r* = –0.70, *p*_Bonferroni_ < 0.05; Fig. 4*I*). Mediation analysis showed that this atypically shorter neural timescale in the left SFG enhanced the A–C–F–B transition, which reduced cognitive rigidity of the ASD+ADHD children and resulted in their ADHD-like cognitive instability (Fig. 4*J*).

These results suggest that pure ADHD and ASD+ADHD comorbidity are underpinned by the different brain state dynamics that are triggered by different local neural activities.

### Bridges between local and global brain dynamics

How can such a local neural activity of a single brain area affect the dynamics of the entire brain activity? We filled this gap by examining the intrinsic neural timescale of the brain networks.

**Figure 5.**
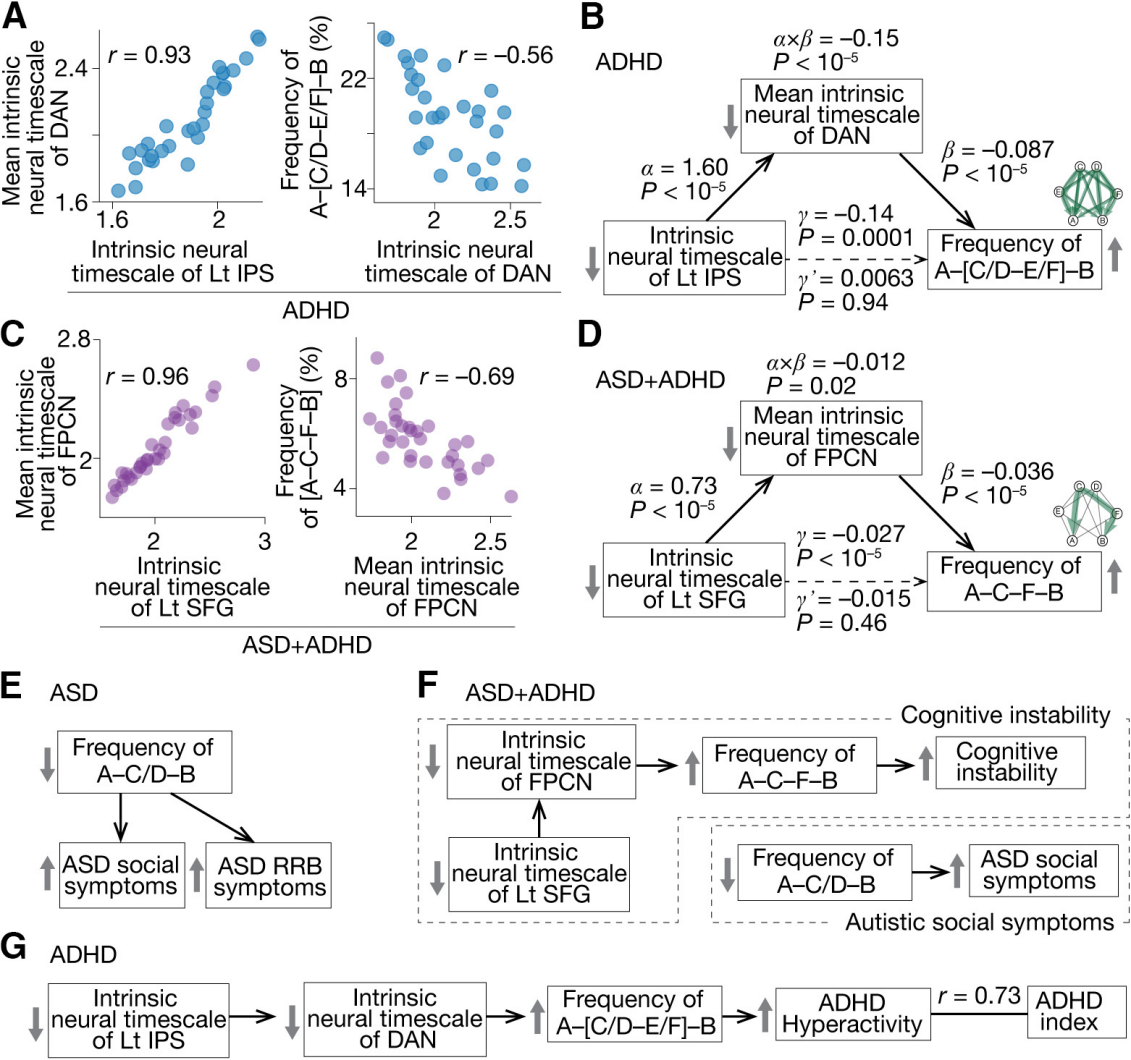
Focal neural activity, brain network activity and whole-brain dynamics. We investigated the mechanisms by which local neural activity affected the whole-brain neural dynamics. ***A***, In the pure ADHD group, the neural timescale of the left inferior parietal sulcus (IPS) was correlated with that of the dorsal attention network (DAN), the parent network of the IPS, which was associated with the frequency of the A–[C/D–E/F]–B transition. ***B***, A mediation analysis showed that the short neural timescale of the left IPS increased the frequency of the A–C–F–B transition by reducing the neural timescale of the DAN. ***C***, In the ASD+ADHD children, the intrinsic neural timescale of the left superior frontal gyrus (SFG) was correlated with that of the frontoparietal control network (FPCN), the parental network of the SFG, which was related to the A–C–F–B transition frequency. ***D***, A mediation analysis demonstrated that the shorter neural timescale of the left SFG enhanced the A–C–F–B transition frequency by decreasing the neural timescale of the FPCN. ***E***, The autistic behavior in the pure ASD children was explained by the atypical reduction in the A–C/D–B transition frequency. ***F***, In the ASD+ADHD children, their ASD symptom was correlated with the atypical decrease in the A–C/D–B transition frequency. Their ADHD-like cognitive instability was induced by the atypically frequent A–C–F–B transition, which was triggered by the unstable activities of FPCN and left SFG. ***G***, The hyperactivity of the pure ADHD children was underpinned by the atypically frequent A–[C/D–E/F]–B transition, which was attributable to the fluctuating activity of the FPCN and left IPS.

In the pure ADHD children, we focused on the dorsal attention network (DAN), to which the left IPS belongs. The mean neural timescale of the DAN was correlated with both that of the left IPS and the A–[C/D–E/F]–B transition frequency (|*r*| > 0.56, *p*_Bonferroni_ < 0.05; Fig. 5*A*). A mediation analysis demonstrated that the fluctuation of the left IPS destabilized the DAN activity, which increased the A–[C/D–E/F]–B transition frequency (Fig. 5*B*).

In the ASD+ADHD group, we examined the mean neural timescale of the frontoparietal control network (FPCN), which includes the left SFG. The neural timescale of the FPCN was associated with both that of the left SFG and the frequency of the A–C–F–B transition (|*r*| > 0.69, *p*_Bonferroni_ < 0.05; Fig. 5*C*). A mediation analysis indicated a link from the atypically short neural timescale of the SFG to the overly frequent A–C–F–B transition via the shorter neural timescale of the FPCN (Fig. 5*D*).

These results indicate that the shorter neural timescales of the focal brain regions, such as the left IPS and SFG, accelerate the relevant brain state transitions by destabilizing the activities of their parent networks.

### Neural dynamics underlying ASD+ADHD comorbidity

In sum, the autistic socio-communicational traits of the ASD+ADHD children were associated with the same infrequent A–C/D–B transition as seen in the pure ASD individuals (Fig. 5*E*,*F*, autistic social symptoms), whereas their ADHD-like cognitive instability was underpinned by unique biological mechanisms that were not linked with the core symptoms of the pure ADHD children. The cognitive instability of the ASD+ADHD comorbidity was correlated with the atypically frequent A–C–F–B transition, which was attributable to the unstable activity of the FPCN and left SFG (Fig. 5*F*, cognitive instability). In contrast, the hyperactivity of the pure ADHD group was grounded on the overly frequent transitions along the A–[C/D–E/F]–B pathway, which was induced by the fluctuating neural activity of the DAN and the left IPS (Fig. 5*G*).

### Confirmatory tests

Qualitatively, the same findings were observed in two independent datasets collected at Kennedy Krieger Institute (KKI) and Oregon Health and Science University (OHSU; Table 2).

First, we confirmed that, in both datasets, the pairwise maximum entropy model was accurately fitted to all the types of participant data (≥87.5%), and the energy landscape analysis identified the same six brain states (States A–F) with the same six local minima (local min *a*–*f*) as in the original results.

**Figure 6.**
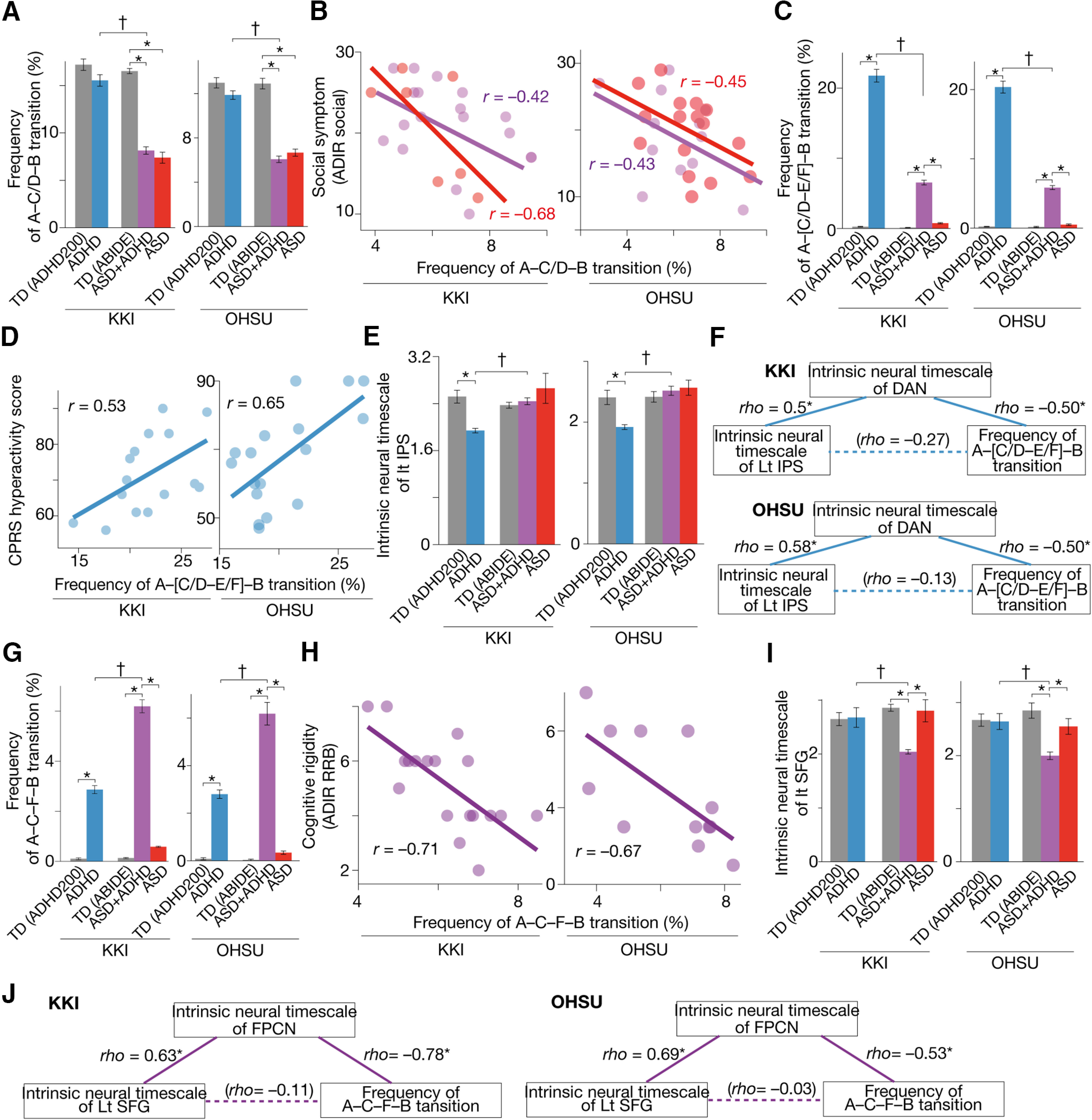
Confirmatory tests. We confirmed that the main findings were qualitatively preserved in two independent datasets: data collected at Kennedy Krieger Institute (KKI) and those recorded at Oregon Health and Science University (OHSU). The pure ASD and ASD+ADHD children had significantly infrequent transitions along the A–C/D–B pathway (***A***), which were correlated with their autistic socio-communicational symptoms (***B***). The frequency of the A–[C/D–E/F]–B transition was atypically higher in the pure ADHD children (***C***) and associated with their hyperactivity tendency (***D***). The intrinsic neural timescale of the left IPS in the pure ADHD children was atypically shorter than controls (***E***) and correlated with the atypically frequent A–[C/D–E/F]–B transition via the unstable activity of the DAN (***F***). The A–C–F–B transition frequency in the ASD+ADHD children was atypically frequent (***G***) and correlated with their cognitive instability (***H***). This atypical A–C–F–B transition frequency was correlated with the atypically shorter neural timescale of the left SFG (***I***) via the fluctuating neural activity of the FPCN (***J***). *p_Bonferroni_ < 0.05, ^†^*p *<* *0.05 for interaction in a two-way ANOVA. The error bars represent the SDs.

The frequency of the A–C/D–B transitions in the pure ASD and ASD+ADHD children was lower than that in the other groups (*p*_Bonferroni_ < 0.05; Fig. 6*A*) and significantly correlated with the severity of their autistic socio-communicational symptoms (*r* ≤ –0.44; Fig. 6*B*).

The A–[C/D–E/F]–B transition frequency in the pure ADHD children was atypically higher than in the other groups (*p*_Bonferroni_ < 0.05; Fig. 6*C*) and predictive of their hyperactivity (*r *≥* *0.53; Fig. 6*D*).

This overly frequent transition in the pure ADHD individuals was linked with the atypically short neural timescale of the left IPS (*t*_(38)_ > 3.7, *p *<* *0 0.05; Fig. 6*E*) via the unstable neural dynamics of the DAN (|ρ| ≥ 0.5, *p *<* *0.05; Fig. 6*F*).

The A–C–F–B transition in the ASD+ADHD children was more frequent than in the other participant groups (*p*_Bonferroni_ < 0.05; Fig. 6*G*) and linked with their cognitive instability (*r* ≤ –0.67; Fig. 6*H*). This frequent transition in the ASD+ADHD condition was associated with the atypically short neural timescale of the left SFG (*t*_(23)_ > 2.8, *p *<* *0 0.05; Fig. 6*I*) via the neural fluctuation of the FPCN (|ρ| ≥ 0.53, *p *<* *0.05; Fig. 6*J*).

Note that the atypical changes in the neural timescales seen in the left IPS and SFG were not detected in exploratory whole-brain analyses of the intrinsic neural timescale, presumably because of the smaller sample sizes of the datasets used in this confirmatory test.

### Associations between ADHD-like hyperactivity and ASD-like RRB

Throughout the above analyses, we assumed that the ADHD-like trait, in particular, hyperactivity, and the ASD-like RRB trait can be located at opposite ends of one dimension representing cognitive rigidity/flexibility. We indirectly confirmed this assumption with a behavioral experiment employing 30 TD adults.

**Figure 7.**
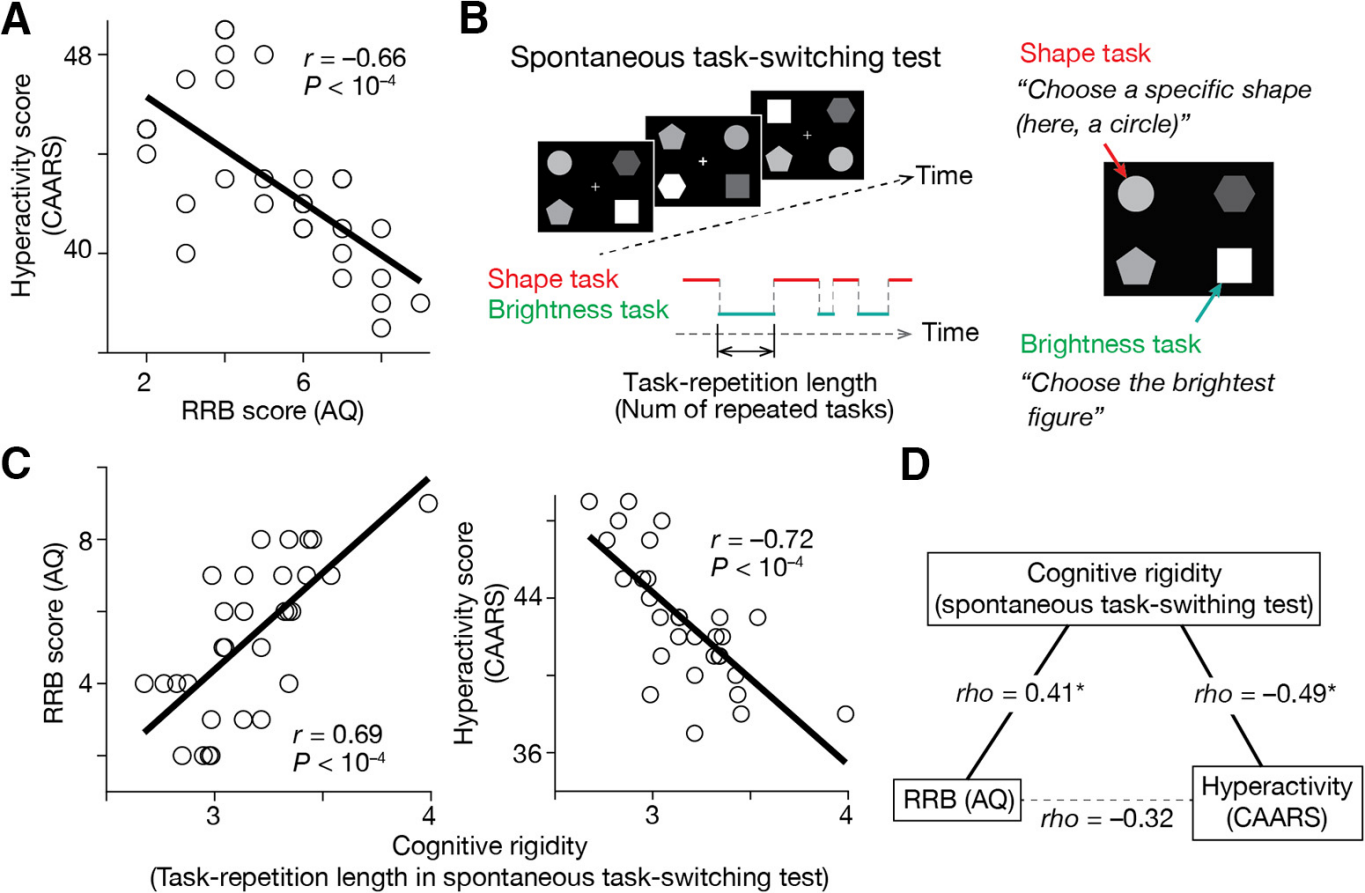
Additional behavioral experiment. The current study assumed that the ADHD-like traits in autistic individuals are related to cognitive overflexibility and inversely correlated with their RRB score. We indirectly examined this assumption with a behavioral experiment employing 30 TD adults. ***A***, We first confirmed a significant negative correlation between their ADHD-like hyperactivity, which was measured by CAARS, and their ASD-like RRB trait, which was calculated as a summation of “Attention switching” and “Attention to details” scores in AQ. ***B***, Second, we quantified the cognitive rigidity, an inverse form of cognitive flexibility, using a spontaneous task-switching test. This test allows us to quantify cognitive rigidity by counting how many same tasks the participants repeated spontaneously. ***C***, The cognitive rigidity was associated with the RRB score in AQ and inversely correlated with the hyperactivity score measured by CAARS. ***D***, A partial correlation analysis indicates that both the RRB score in AQ and hyperactivity score in CAARS would be behavioral manifestations of cognitive rigidity. *p_Bonferroni_ < 0.05.

First, we asked the participants to complete questionnaires to evaluate their ADHD traits (CAARS; Conners et al., 1999) and those for the examination of their ASD tendency (AQ; Baron-Cohen et al., 2001; Wakabayashi et al., 2006) and found a significant inverse correlation between the hyperactivity score in CAARS and the RRB score (“Attention switching” + “Attention to details”) in AQ (*r* = –0.66, *p *<* *10^−4^; Fig. 7*A*).

Second, the participants underwent a spontaneous task-switching test, which is thought to allow us to quantify their cognitive rigidity (Arrington and Logan, 2004; Poljac et al., 2012; Watanabe et al., 2019a; Fig. 7*B*). We found that the task-repetition length observed in the test was correlated with the RRB score in AQ (*r *=* *0.69, *p *<* *10^−4^; Fig. 7*C*, left panel) and negatively associated with the hyperactivity score in CAARS (*r* = –0.72, *p *<* *10^−4^; Fig. 7*C*, right panel).

Moreover, a partial correlation analysis indicated that both the RRB score in AQ and the hyperactivity score in CAARS are manifestations of cognitive rigidity/flexibility (Fig. 7*D*).

Albeit this experiment employed TD adults, these observations indicate that we could put the ASD-like RRB score and ADHD-like hyperactivity at opposite ends of cognitive rigidity, which indirectly support our usage of the ADI-R RRB score to evaluate ADHD-like hyperactivity in the ASD+ADHD children.

## Discussion

This study has directly compared the brain dynamics underlying the high-functioning ASD+ADHD, pure ASD and pure ADHD children and found that the ASD+ADHD comorbidity is not a mere overlap of the two prevalent neurodevelopmental disorders. The autistic socio-communicational traits of the ASD+ADHD children were explained by the same neural rigidity as that of the pure ASD individuals. In contrast, their ADHD-like cognitive instability was attributable to an atypically frequent brain state transition and unstable local neural activity, neither of which was associated with any core symptom of the pure ADHD children. These findings have uncovered the unique brain mechanisms underpinning the ASD+ADHD comorbidity and, in particular, indicated that ADHD-like cognitive instability seen in ASD+ADHD children may have to be treated as a distinct neurodevelopmental condition.

One of the limitations of this study is in the manner of evaluating the ADHD-like traits in the ASD+ADHD children. Because the ABIDE project focuses on autism, we could not obtain CPRS scores for the ASD+ADHD children. Instead, their cognitive instability was inferred based on the RRB score in the ADI-R system. Such an inference is based on previous findings on (1) close links between the ADHD symptoms and cognitive flexibility/instability (Semrud-Clikeman et al., 2010; Das et al., 2014) and (2) significant inverse correlations between the cognitive instability and the RRB score in clinical tests (Lopez et al., 2005; Watanabe et al., 2019a; Cissne et al., 2022). Although this inference was indirectly supported by the behavioral experiment employing TD individuals (Fig. 7), future studies may have to use ADHD-specific behavioral indices to measure such ADHD-like traits of ASD+ADHD individuals.

Psychologically, the current findings allow us to speculate cognitive differences between the hyperactivity of pure ADHD and cognitive instability of the ASD+ADHD comorbidity. Given that the DAN is closely linked to attention selection and generation of attentional sets (Spadone et al., 2015; Corbetta and Shulman, 2002), the close link between the ADHD hyperactivity and unstable DAN activity implies that the hyperactivity of pure ADHD could represent insufficient control of top-down attention. In contrast, considering that the FPCN is involved with the coordination of multiple functional systems in the brain (Cole et al., 2012, 2013; Cocuzza et al., 2020), the correlation between the cognitive instability of the ASD+ADHD children and unstable FPCN activity indicates the cognitive instability of ASD+ADHD children may not be a direct consequence of atypical attention but represent the insufficient integration of various neural information.

Clinically, this study implies the possibility of new noninvasive treatments for ASD, ADHD, and ASD+ADHD comorbidity. For example, as demonstrated in our recent study (Watanabe, 2021), some behavioral traits can be changed by a brain-state-driven neural stimulation system, in which we can modulate the structure of the energy landscape and modify relevant behaviors. Therefore, the hyperactivity of pure ADHD may be mitigable by means of stabilizing states A and B and destabilizing states E and F.

In addition, the current findings may indicate the necessity to re-evaluate the conventional treatments for high-functioning adults with both ASD and ADHD. In particular, given that the ADHD-like traits of ASD+ADHD individuals and those of pure ADHD patients are underpinned by different biological mechanisms (Fig. 5*F*,*G*), we may have to re-examine the medication on ADHD symptoms seen in the comorbid condition. In fact, some previous studies reported that mainstream medication for pure ADHD, such as methylphenidate, did not have as sufficient effects on ADHD-like behaviors of ASD+ADHD individuals as those of pure ADHD children (M.J. Murray, 2010; Joshi and Wilens, 2022). One research using functional near-infrared spectroscopy even found a significant difference in the whole-brain neural responses to methylphenidate between ASD+ADHD and pure ADHD children (Sutoko et al., 2019). Considering these previous findings along with the current observation, we might have to search for novel interventions to mitigate ADHD-like behaviors of the ASD+ADHD comorbidity.

This neurobiological distinctiveness of the ASD+ADHD condition is also consistent with a recent systematic review of behavioral literature on the comorbidity (Benallie et al., 2021): the review suggested that, compared with pure ADHD children, ASD+ADHD individuals have unique executive functions that are particularly related to planning and organizing. This behavioral difference may be attributable to the distinct properties between the DAN and FPCN.

The association between ASD symptoms and rigid brain state dynamics is consistent with and expands our previous findings (Watanabe and Rees, 2017). The prior work found that high-functioning autistic adults and typically developing controls shared the same two stable brain states (major states) and two relatively unstable ones (minor states). In contrast, the frequency of the transitions between the major states via one of the minor states was significantly reduced in the ASD adults, and such atypical reduction predicted the severity of their ASD symptoms. Given the qualitative similarity between the major states in the prior work and the states A and B in this study and between the minor states in the prior work and the states C and D in this research, the significant correlation between the ASD symptoms and the A–C/D–B transition frequency can be seen as additional evidence for our previous observations. Also, the current finding shows that this brain-symptom association is not limited to ASD adults but is applicable to children with autistic traits.

Despite intensive neuroimaging research on pure ADHD (Dickstein et al., 2006; Castellanos et al., 2002; Rubia et al., 2010; Castellanos and Proal, 2012; Cortese et al., 2012; Hart et al., 2012, 2013; Rajagopal et al., 2022), the global brain state dynamics of the neurodevelopmental condition were little understood. One study examined such macroscopic neural dynamics by applying a version of energy landscape analysis (Jeong et al., 2021), but the main purpose of the study was the validation of its analysis method but not the investigation of biological mechanisms behind ADHD. In addition, the prior work focused on the default mode network and executive control network but did not examine the brain-wide neural dynamics. Considering this, the current findings may be one of the first observations on the global brain state dynamics underpinning ADHD symptoms in humans.

Using the two data-driven analyses, the current study identified the distinct neural dynamics underpinning the ASD+ADHD comorbidity. The autistic social symptoms of the ASD+ADHD children were associated with similar neural bases as those of the pure ASD individuals, whereas their ADHD-like cognitive instability was governed by the unique global and local brain dynamics compared with the pure ADHD children. These observations indicate that “ASD+ADHD” is not a simple overlap of ASD and ADHD, and in particular, its cognitive instability would represent a distinct disorder and need unique treatments. The current approach focusing on the global and local neural dynamics could provide a new perspective for a comprehensive biological understanding of multiple neuropsychiatric disorders.

## Synthesis

Reviewing Editor: Christoph Michel, Universite de Geneve Decisions are customarily a result of the Reviewing Editor and the peer reviewers coming together and discussing their recommendations until a consensus is reached. When revisions are invited, a fact-based synthesis statement explaining their decision and outlining what is needed to prepare a revision will be listed below. The following reviewer(s) agreed to reveal their identity: Matthew Belmonte. Note: If this manuscript was transferred from JNeurosci and a decision was made to accept the manuscript without peer review, a brief statement to this effect will instead be what is listed below.

One of the original reviewers of the J. Neuroscience submission accepted to re-review the revised manuscript. Given his/her positive evaluation I decided that only minor revisions are required before the paper can be accepted for publication in eNeuro. The comments of the reviewer are appended below.

The authors’ rebuttal is well taken. It doesn’t actually challenge the essence of this reviewer’s objection- only a prospectively collected data set can do that!- but at least the manuscript text is now more cautious. It might be prudent also to introduce this caution in the abstract. (Some room in the abstract could be freed by deleting unnecessary phrases “Here, to address this situation”, “Regarding autistic symptoms”, “that was”, “that were”, and both instances of “neural”.)

Similarly, the title might be rendered most descriptive of this study’s findings were the functional anatomical word ‘frontoparietal’ inserted after the word ‘distinct’, viz., “Distinct frontoparietal brain dynamics underlying the co-occurrence of autism and ADHD”-- just a suggestion, take it or leave it.

Reagrding the speculation “the current findings may indicate the necessity to re-evaluate the conventional treatments for high-functioning adults with ASD and ADHD”, these sources might be deemed germane- or might not, at the authors’ option:

10.1007/s11920-010-0145-3

10.1016/j.chc.2022.03.012

10.3389/fnhum.2019.00007

These suggestions are provided for the authors’ consideration in preparing their final copy, and not for re-review.

Minor correction on page 6, line 15: Instead of “could not be”, ought this phrase to be “is” or “might be”?

## References

[B49] ADHD-200 Consortium (2012) The ADHD-200 Consortium: a model to advance the translational potential of neuroimaging in clinical neuroscience. Frontiers Syst Neurosci 6:62. 10.3389/fnsys.2012.00062 PMC3433679 22973200

[B1] Aoki Y, Yoncheva YN, Chen B, Nath T, Sharp D, Lazar M, Velasco P, Milham MP, Martino AD (2017) Association of white matter structure with autism spectrum disorder and attention-deficit/hyperactivity disorder. JAMA Psychiatry 4:1120–1128. 10.1001/jamapsychiatry.2017.2573 28877317 PMC5710226

[B2] American Psychiatric Association (2022) Diagnostic and statistical manual of mental disorders, DSM-5-TR. Washington, DC: Amer Psychiatric Pub Inc.

[B3] Arrington CM, Logan GD (2004) The cost of a voluntary task switch. Psychol Sci 15:610–615. 10.1111/j.0956-7976.2004.00728.x 15327632

[B4] Baldassano C, Chen J, Zadbood A, Pillow JW, Hasson U, Norman KA (2017) Discovering event structure in continuous narrative perception and memory. Neuron 95:709–721.e5. 10.1016/j.neuron.2017.06.041 28772125 PMC5558154

[B5] Baron-Cohen S, Wheelwright S, Skinner R, Martin J, Clubley E (2001) The autism-spectrum quotient (AQ): evidence from Asperger syndrome/high-functioning autism, males and females, scientists and mathematicians. J Autism Dev Disord 31:5–17. 10.1023/a:1005653411471 11439754

[B6] Benallie KJ, McClain MB, Bakner KE, Roanhorse T, Ha J (2021) Executive functioning in children with ASD + ADHD and ASD + ID: a systematic review. Res Autism Spect Dis 86:101807. 10.1016/j.rasd.2021.101807

[B7] Berenguer C, Rosello B, Leader G (2018) A review of executive functions in autism spectrum disorder and attention deficit hyperactivity disorder. J Educ Dev Psychology 8:107. 10.5539/jedp.v8n2p107

[B8] Boedhoe PSW, et al. (2020) Subcortical brain volume, regional cortical thickness, and cortical surface area across disorders: findings from the ENIGMA ADHD, ASD, and OCD working groups. Am J Psychiatry 177:834–843. 10.1176/appi.ajp.2020.19030331 32539527 PMC8296070

[B9] Bourgeron T (2015) From the genetic architecture to synaptic plasticity in autism spectrum disorder. Nat Rev Neurosci 16:551–563. 10.1038/nrn3992 26289574

[B10] Brierley NJ, McDonnell CG, Parks KMA, Schulz SE, Dalal TC, Kelley E, Anagnostou E, Nicolson R, Georgiades S, Crosbie J, Schachar R, Liu X, Stevenson RA (2021) Factor structure of repetitive behaviors across autism spectrum disorder and attention-deficit/hyperactivity disorder. J Autism Dev Disord 51:3391–3400. 10.1007/s10803-020-04800-0 33236274

[B11] Bühler E, Bachmann C, Goyert H, Heinzel-Gutenbrunner M, Kamp-Becker I (2011) Differential diagnosis of autism spectrum disorder and attention deficit hyperactivity disorder by means of inhibitory control and ‘theory of mind.’ J Autism Dev Disord 41:1718–1726. 10.1007/s10803-011-1205-1 21373957

[B12] Cai W, Chen T, Szegletes L, Supekar K, Menon V (2018) Aberrant time-varying cross-network interactions in children with attention-deficit/hyperactivity disorder and the relation to attention deficits. Biol Psychiatry Cogn Neurosci Neuroimaging 3:263–273. 10.1016/j.bpsc.2017.10.005 29486868 PMC5833018

[B13] Castellanos FX, Proal E (2012) Large-scale brain systems in ADHD: beyond the prefrontal–striatal model. Trends Cogn Sci 16:17–26. 10.1016/j.tics.2011.11.007 22169776 PMC3272832

[B14] Castellanos FX, Lee PP, Sharp W, Jeffries NO, Greenstein DK, Clasen LS, Blumenthal JD, James RS, Ebens CL, Walter JM, Zijdenbos A, Evans AC, Giedd JN, Rapoport JL (2002) Developmental trajectories of brain volume abnormalities in children and adolescents with attention-deficit/hyperactivity disorder. JAMA 288:1740–1748. 10.1001/jama.288.14.1740 12365958

[B15] Chantiluke K, Christakou A, Murphy CM, Giampietro V, Daly EM, Ecker C, Brammer M, Murphy DG; MRC AIMS Consortium; Rubia K (2014) Disorder-specific functional abnormalities during temporal discounting in youth with attention deficit hyperactivity disorder (ADHD), autism and comorbid ADHD and autism. Psychiatry Res 223:113–120. 10.1016/j.pscychresns.2014.04.006 24929553

[B16] Chen J, Hasson U, Honey CJ (2015) Processing timescales as an organizing principle for primate cortex. Neuron 88:244–246. 10.1016/j.neuron.2015.10.010 26494274

[B17] Christakou A, Murphy CM, Chantiluke K, Cubillo AI, Smith AB, Giampietro V, Daly E, Ecker C, Robertson D, consortium MA; MRC AIMS consortium; Murphy DG, Rubia K (2013) Erratum: disorder-specific functional abnormalities during sustained attention in youth with attention deficit hyperactivity disorder (ADHD) and with autism. Mol Psychiatry 18:264–264. 10.1038/mp.2012.41 PMC3554878 22290121

[B18] Cissne MN, Kester LE, Gunn AJM, Bodner KE, Miles JH, Christ SE (2022) Brief report: a preliminary study of the relationship between repetitive behaviors and concurrent executive function demands in children with autism spectrum disorder. J Autism Dev Disord 52:1896–1902. 10.1007/s10803-021-05071-z 34009548

[B19] Cocuzza CV, Ito T, Schultz D, Bassett DS, Cole MW (2020) Flexible coordinator and switcher hubs for adaptive task control. J Neurosci 40:6949–6968. 10.1523/JNEUROSCI.2559-19.2020 32732324 PMC7470914

[B20] Cole MW, Yarkoni T, Repovs G, Anticevic A, Braver TS (2012) Global connectivity of prefrontal cortex predicts cognitive control and intelligence. J Neurosci 32:8988–8999. 10.1523/JNEUROSCI.0536-12.2012 22745498 PMC3392686

[B21] Cole MW, Reynolds JR, Power JD, Repovs G, Anticevic A, Braver TS (2013) Multi-task connectivity reveals flexible hubs for adaptive task control. Nat Neurosci 16:1348–1355. 10.1038/nn.3470 23892552 PMC3758404

[B22] Colombi C, Ghaziuddin M (2017) Neuropsychological characteristics of children with mixed autism and ADHD. Autism Res Treat 2017:5781781. 10.1155/2017/5781781 28811938 PMC5547727

[B23] Conners CK, Erhardt D, Epstein JN, Parker JDA, Sitarenios G, Sparrow E (1999) Self-ratings of ADHD symptoms in adults I: factor structure and normative data. J Atten Disord 3:141–151. 10.1177/108705479900300303

[B24] Cooper M, Martin J, Langley K, Hamshere M, Thapar A (2014) Autistic traits in children with ADHD index clinical and cognitive problems. Eur Child Adolesc Psychiatry 23:23–34. 10.1007/s00787-013-0398-6 23616179 PMC3899449

[B25] Corbett BA, Constantine LJ, Hendren R, Rocke D, Ozonoff S (2009) Examining executive functioning in children with autism spectrum disorder, attention deficit hyperactivity disorder and typical development. Psychiatry Res 166:210–222. 10.1016/j.psychres.2008.02.005 19285351 PMC2683039

[B26] Corbetta M, Shulman GL (2002) Control of goal-directed and stimulus-driven attention in the brain. Nat Rev Neurosci 3:201–215. 10.1038/nrn755 11994752

[B27] Cortese S, Kelly C, Chabernaud C, Proal E, Martino AD, Milham MP, Castellanos FX (2012) Toward systems neuroscience of ADHD: a meta-analysis of 55 fMRI studies. Am J Psychiatry 169:1038–1055. 10.1176/appi.ajp.2012.11101521 22983386 PMC3879048

[B28] Craig F, Lamanna AL, Margari F, Matera E, Simone M, Margari L (2015) Overlap between autism spectrum disorders and attention deficit hyperactivity disorder: searching for distinctive/common clinical features. Autism Res 8:328–337. 10.1002/aur.1449 25604000 PMC4654237

[B29] Craig F, Margari F, Legrottaglie AR, Palumbi R, de Giambattista C, Margari L (2016) A review of executive function deficits in autism spectrum disorder and attention-deficit/hyperactivity disorder. Neuropsychiatr Dis Treat 12:1191–1202. 10.2147/NDT.S104620 27274255 PMC4869784

[B30] Cross-Disorder Group of the Psychiatric Genomics Consortium, et al. (2013) Genetic relationship between five psychiatric disorders estimated from genome-wide SNPs. Nat Genet 45:984–994. 23933821 10.1038/ng.2711 PMC3800159

[B31] Curtin P, Neufeld J, Curtin A, Arora M, Bölte S (2022) Altered periodic dynamics in the default mode network in autism and attention-deficit/hyperactivity disorder. Biol Psychiatry 91:956–966. 10.1016/j.biopsych.2022.01.010 35227462 PMC9119910

[B32] Dajani DR, Llabre MM, Nebel MB, Mostofsky SH, Uddin LQ (2016) Heterogeneity of executive functions among comorbid neurodevelopmental disorders. Sci Rep 6:36566. 10.1038/srep36566 27827406 PMC5101520

[B33] Dajani DR, Burrows CA, Odriozola P, Baez A, Nebel MB, Mostofsky SH, Uddin LQ (2019) Investigating functional brain network integrity using a traditional and novel categorical scheme for neurodevelopmental disorders. Neuroimage Clin 21:101678. 10.1016/j.nicl.2019.101678 30708240 PMC6356009

[B34] Das D, Cherbuin N, Easteal S, Anstey KJ (2014) Attention deficit/hyperactivity disorder symptoms and cognitive abilities in the late-life cohort of the PATH through life study. PLoS One 9:e86552. 10.1371/journal.pone.0086552 24489743 PMC3904910

[B35] de Lacy N, Calhoun VD (2018) Dynamic connectivity and the effects of maturation in youth with attention deficit hyperactivity disorder. Netw Neurosci 3:195–216. 10.1162/netn_a_00063 30793080 PMC6372020

[B36] Demetriou EA, Lampit A, Quintana DS, Naismith SL, Song YJC, Pye JE, Hickie I, Guastella AJ (2018) Autism spectrum disorders: a meta-analysis of executive function. Mol Psychiatry 23:1198–1204. 10.1038/mp.2017.75 28439105 PMC5984099

[B37] Dickstein SG, Bannon K, Castellanos FX, Milham MP (2006) The neural correlates of attention deficit hyperactivity disorder: an ALE meta‐analysis. J Child Psychol Psychiatry 47:1051–1062. 10.1111/j.1469-7610.2006.01671.x 17073984

[B38] Di Martino A, et al. (2014) The autism brain imaging data exchange: towards a large-scale evaluation of the intrinsic brain architecture in autism. Mol Psychiatr 19:659–667. 10.1038/mp.2013.78 PMC4162310 23774715

[B39] Di Martino A, et al. (2017) Enhancing studies of the connectome in autism using the autism brain imaging data exchange II. Sci Data 4:170010. 28291247 10.1038/sdata.2017.10 PMC5349246

[B40] Ezaki T, Watanabe T, Ohzeki M, Masuda N (2017) Energy landscape analysis of neuroimaging data. Philos Trans A Math Phys Eng Sci 375:20160287. 10.1098/rsta.2016.0287 28507232 PMC5434078

[B41] Gargaro BA, Rinehart NJ, Bradshaw JL, Tonge BJ, Sheppard DM (2011) Autism and ADHD: how far have we come in the comorbidity debate? Neurosci Biobehav Rev 35:1081–1088. 10.1016/j.neubiorev.2010.11.002 21093480

[B42] Girvan M, Newman MEJ (2002) Community structure in social and biological networks. Proc Natl Acad Sci U S A 99:7821–7826. 10.1073/pnas.122653799 12060727 PMC122977

[B43] Hansen JY, et al. (2022) Local molecular and global connectomic contributions to cross-disorder cortical abnormalities. Nat Commun 13:4682. 10.1038/s41467-022-32420-y 35948562 PMC9365855

[B44] Harikumar A, Evans DW, Dougherty CC, Carpenter KLH, Michael AM (2021) A review of the default mode network in autism spectrum disorders and attention deficit hyperactivity disorder. Brain Connect 11:253–263. 10.1089/brain.2020.0865 33403915 PMC8112713

[B45] Hart H, Radua J, Mataix-Cols D, Rubia K (2012) Meta-analysis of fMRI studies of timing in attention-deficit hyperactivity disorder (ADHD). Neurosci Biobehav Rev 36:2248–2256. 10.1016/j.neubiorev.2012.08.003 22922163

[B46] Hart H, Radua J, Nakao T, Mataix-Cols D, Rubia K (2013) Meta-analysis of functional magnetic resonance imaging studies of inhibition and attention in attention-deficit/hyperactivity disorder: exploring task-specific, stimulant medication, and age effects. JAMA Psychiatry 70:185–198. 10.1001/jamapsychiatry.2013.277 23247506

[B47] Hasson U, Yang E, Vallines I, Heeger DJ, Rubin N (2008) A hierarchy of temporal receptive windows in human cortex. J Neurosci 28:2539–2550. 10.1523/JNEUROSCI.5487-07.2008 18322098 PMC2556707

[B48] Hasson U, Chen J, Honey CJ (2015) Hierarchical process memory: memory as an integral component of information processing. Trends Cogn Sci 19:304–313. 10.1016/j.tics.2015.04.006 25980649 PMC4457571

[B50] Honey CJ, Thesen T, Donner TH, Silbert LJ, Carlson CE, Devinsky O, Doyle WK, Rubin N, Heeger DJ, Hasson U (2012) Slow cortical dynamics and the accumulation of information over long timescales. Neuron 76:423–434. 10.1016/j.neuron.2012.08.011 23083743 PMC3517908

[B51] Hong SJ, de Wael RV, Bethlehem RAI, Lariviere S, Paquola C, Valk SL, Milham MP, Martino AD, Margulies DS, Smallwood J, Bernhardt BC (2019) Atypical functional connectome hierarchy in autism. Nat Commun 10:1022. 10.1038/s41467-019-08944-1 30833582 PMC6399265

[B52] Hoogman M, et al. (2022) Consortium neuroscience of attention deficit/hyperactivity disorder and autism spectrum disorder: the ENIGMA adventure. Hum Brain Mapp 43:37–55. 10.1002/hbm.25029 32420680 PMC8675410

[B53] Hours C, Recasens C, Baleyte JM (2022) ASD and ADHD comorbidity: what are we talking about? Front Psychiatry 13:837424. 35295773 10.3389/fpsyt.2022.837424 PMC8918663

[B54] Jeong SO, Kang J, Pae C, Eo J, Park SM, Son J, Park H-J (2021) Empirical Bayes estimation of pairwise maximum entropy model for nonlinear brain state dynamics. Neuroimage 244:118618. 10.1016/j.neuroimage.2021.118618 34571159

[B55] Joshi G, Wilens TE (2022) Pharmacotherapy of attention-deficit/hyperactivity disorder in individuals with autism spectrum disorder. Child Adolesc Psychiatr Clin N Am 31:449–468. 10.1016/j.chc.2022.03.012 35697395

[B56] Joshi G, Faraone SV, Wozniak J, Tarko L, Fried R, Galdo M, Furtak SL, Biederman J (2017) Symptom profile of ADHD in youth with high-functioning autism spectrum disorder: a comparative study in psychiatrically referred populations. J Atten Disord 21:846–855. 10.1177/1087054714543368 25085653 PMC4312732

[B57] Kaat AJ, Gadow KD, Lecavalier L (2013) Psychiatric symptom impairment in children with autism spectrum disorders. J Abnorm Child Psychol 41:959–969. 10.1007/s10802-013-9739-7 23605958

[B58] Kaboodvand N, Iravani B, Fransson P (2020) Dynamic synergetic configurations of resting-state networks in ADHD. Neuroimage 207:116347. 10.1016/j.neuroimage.2019.116347 31715256

[B59] Kang J, Pae C, Park H-J (2017) Energy landscape analysis of the subcortical brain network unravels system properties beneath resting state dynamics. Neuroimage 149:153–164. 10.1016/j.neuroimage.2017.01.075 28159684

[B60] Kotte A, Joshi G, Fried R, Uchida M, Spencer A, Woodworth KY, Kenworthy T, Faraone SV, Biederman J (2013) Autistic traits in children with and without ADHD. Pediatrics 132:e612–e622. 10.1542/peds.2012-3947 23979086 PMC3876754

[B61] Kuntsi J, Klein C (2011) Behavioral neuroscience of attention deficit hyperactivity disorder and its treatment. Curr Top Behav Neurosci 9:67–91.

[B62] Lake EMR, Finn ES, Noble SM, Vanderwal T, Shen X, Rosenberg MD, Spann MN, Chun MM, Scheinost D, Constable RT (2019) The functional brain organization of an individual allows prediction of measures of social abilities transdiagnostically in autism and attention-deficit/hyperactivity disorder. Biol Psychiatry 86:315–326. 10.1016/j.biopsych.2019.02.019 31010580 PMC7311928

[B63] Lau-Zhu A, Fritz A, McLoughlin G (2019) Overlaps and distinctions between attention deficit/hyperactivity disorder and autism spectrum disorder in young adulthood: systematic review and guiding framework for EEG-imaging research. Neurosci Biobehav Rev 96:93–115. 10.1016/j.neubiorev.2018.10.009 30367918 PMC6331660

[B64] Lawson RA, Papadakis AA, Higginson CI, Barnett JE, Wills MC, Strang JF, Wallace GL, Kenworthy L (2015) Everyday executive function impairments predict comorbid psychopathology in autism spectrum and attention deficit hyperactivity disorders. Neuropsychology 29:445–453. 10.1037/neu0000145 25313979

[B65] Lim L, Marquand A, Cubillo AA, Smith AB, Chantiluke K, Simmons A, Mehta M, Rubia K (2013) Disorder-specific predictive classification of adolescents with attention deficit hyperactivity disorder (ADHD) relative to autism using structural magnetic resonance imaging. PLoS One 8:e63660. 10.1371/journal.pone.0063660 23696841 PMC3656087

[B66] Lopez BR, Lincoln AJ, Ozonoff S, Lai Z (2005) Examining the relationship between executive functions and restricted, repetitive symptoms of autistic disorder. J Autism Dev Disord 35:445–460. 10.1007/s10803-005-5035-x 16134030

[B67] Martin J, Cooper M, Hamshere ML, Pocklington A, Scherer SW, Kent L, Gill M, Owen MJ, Williams N, O’Donovan MC, Thapar A, Holmans P (2014) Biological overlap of attention-deficit/hyperactivity disorder and autism spectrum disorder: evidence from copy number variants. J Am Acad Child Adolesc Psychiatry 53:761–770.e26. 10.1016/j.jaac.2014.03.004 24954825 PMC4074351

[B68] Massen CP, Doye JPK (2005) Identifying communities within energy landscapes. Phys Rev E Stat Nonlin Soft Matter Phys 71:e046101. 10.1103/PhysRevE.71.046101 15903720

[B69] Mayes SD, Calhoun SL, Mayes RD, Molitoris S (2012) Autism and ADHD: overlapping and discriminating symptoms. Res Autism Spect Dis 6:277–285. 10.1016/j.rasd.2011.05.009

[B70] Mellahn OJ, Knott R, Tiego J, Kallady K, Williams K, Bellgrove MA, Johnson BP (2022) Understanding the diversity of pharmacotherapeutic management of ADHD with co-occurring autism: an Australian cross-sectional survey. Frontiers Psychiatry 13:914668. 10.3389/fpsyt.2022.914668 PMC9271966 35832595

[B71] Murray JD, Bernacchia A, Freedman DJ, Romo R, Wallis JD, Cai X, Padoa-Schioppa C, Pasternak T, Seo H, Lee D, Wang XJ (2014) A hierarchy of intrinsic timescales across primate cortex. Nat Neurosci 17:1661–1663. 10.1038/nn.3862 25383900 PMC4241138

[B72] Murray MJ (2010) Attention-deficit/hyperactivity disorder in the context of autism spectrum disorders. Curr Psychiatry Rep 12:382–388. 10.1007/s11920-010-0145-3 20694583

[B73] Nickel K, Elst LT van, Manko J, Unterrainer J, Rauh R, Klein C, Endres D, Kaller CP, Mader I, Riedel A, Biscaldi M, Maier S (2018) Inferior frontal gyrus volume loss distinguishes between autism and (comorbid) attention-deficit/hyperactivity disorder—a FreeSurfer analysis in children. Front Psychiatry 9:521. 30405459 10.3389/fpsyt.2018.00521 PMC6206215

[B74] Ohta H, Aoki YY, Itahashi T, Kanai C, Fujino J, Nakamura M, Kato N, Hashimoto R (2020) White matter alterations in autism spectrum disorder and attention-deficit/hyperactivity disorder in relation to sensory profile. Mol Autism 11:77. 33070774 10.1186/s13229-020-00379-6 PMC7570037

[B75] Ozonoff S, Jensen J (1999) Brief report: specific executive function profiles in three neurodevelopmental disorders. J Autism Dev Disord 29:171–177. 10.1023/a:1023052913110 10382139

[B76] Poldrack RA (2006) Can cognitive processes be inferred from neuroimaging data? Trends Cogn Sci 10:59–63. 10.1016/j.tics.2005.12.004 16406760

[B77] Poljac E, Poljac E, Yeung N (2012) Cognitive control of intentions for voluntary actions in individuals with a high level of autistic traits. J Autism Dev Disord 42:2523–2533. 10.1007/s10803-012-1509-9 22434281 PMC3490069

[B78] Rajagopal VM, et al. (2022) Differences in the genetic architecture of common and rare variants in childhood, persistent and late-diagnosed attention-deficit hyperactivity disorder. Nat Genet 54:1117–1124. 10.1038/s41588-022-01143-7 35927488 PMC10028590

[B79] Rommelse NNJ, Geurts HM, Franke B, Buitelaar JK, Hartman CA (2011) A review on cognitive and brain endophenotypes that may be common in autism spectrum disorder and attention-deficit/hyperactivity disorder and facilitate the search for pleiotropic genes. Neurosci Biobehav Rev 35:1363–1396. 10.1016/j.neubiorev.2011.02.015 21382410

[B80] Ronald A, Simonoff E, Kuntsi J, Asherson P, Plomin R (2008) Evidence for overlapping genetic influences on autistic and ADHD behaviours in a community twin sample. J Child Psychol Psychiatry 49:535–542. 10.1111/j.1469-7610.2007.01857.x 18221348

[B81] Rong Y, Yang C-J, Jin Y, Wang Y (2021) Prevalence of attention-deficit/hyperactivity disorder in individuals with autism spectrum disorder: a meta-analysis. Res Autism Spect Dis 83:101759. 10.1016/j.rasd.2021.101759

[B82] Roy D, Uddin LQ (2021) Atypical core-periphery brain dynamics in autism. Netw Neurosci 5:295–321. 10.1162/netn_a_00181 34189366 PMC8233106

[B83] Rubia K, Halari R, Cubillo A, Mohammad A, Scott S, Brammer M (2010) Disorder‐specific inferior prefrontal hypofunction in boys with pure attention‐deficit/hyperactivity disorder compared to boys with pure conduct disorder during cognitive flexibility. Hum Brain Mapp 31:1823–1833. 10.1002/hbm.20975 20205245 PMC6870768

[B84] Runyan CA, Piasini E, Panzeri S, Harvey CD (2017) Distinct timescales of population coding across cortex. Nature 548:92–96. 10.1038/nature23020 28723889 PMC5859334

[B85] Sáenz AA, Septier M, Schuerbeek PV, Baijot S, Deconinck N, Defresne P, Delvenne V, Passeri G, Raeymaekers H, Salvesen L, Victoor L, Villemonteix T, Willaye E, Peigneux P, Massat I (2020) ADHD and ASD: distinct brain patterns of inhibition-related activation? Transl Psychiat 10:24. 10.1038/s41398-020-0707-z PMC7026183 32066671

[B86] Safar K, Vandewouw MM, Pang EW, de Villa K, Crosbie J, Schachar R, Iaboni A, Georgiades S, Nicolson R, Kelley E, Ayub M, Lerch JP, Anagnostou E, Taylor MJ (2022) Shared and distinct patterns of functional connectivity to emotional faces in autism spectrum disorder and attention-deficit/hyperactivity disorder children. Front Psychol 13:826527. 10.3389/fpsyg.2022.826527 35356352 PMC8959934

[B87] Saggar M, Shine JM, Liégeois R, Dosenbach NUF, Fair D (2022) Precision dynamical mapping using topological data analysis reveals a hub-like transition state at rest. Nat Commun 13:4791. 10.1038/s41467-022-32381-2 35970984 PMC9378660

[B88] Salazar F, Baird G, Chandler S, Tseng E, O’sullivan T, Howlin P, Pickles A, Simonoff E (2015) Co-occurring psychiatric disorders in preschool and elementary school-aged children with autism spectrum disorder. J Autism Dev Disord 45:2283–2294. 10.1007/s10803-015-2361-5 25737019

[B89] Satterstrom FK, et al. (2019) Autism spectrum disorder and attention deficit hyperactivity disorder have a similar burden of rare protein-truncating variants. Nat Neurosci 22:1961–1965. 10.1038/s41593-019-0527-8 31768057 PMC6884695

[B90] Semrud-Clikeman M, Walkowiak J, Wilkinson A, Butcher B (2010) Executive functioning in children with Asperger syndrome, ADHD-combined type, ADHD-predominately inattentive type, and controls. J Autism Dev Disord 40:1017–1027. 10.1007/s10803-010-0951-9 20140638

[B91] Sergeant JA, Geurts H, Oosterlaan J (2002) How specific is a deficit of executive functioning for attention-deficit/hyperactivity disorder? Behav Brain Res 130:3–28. 10.1016/s0166-4328(01)00430-2 11864714

[B92] Shappell HM, Duffy KA, Rosch KS, Pekar JJ, Mostofsky SH, Lindquist MA, Cohen JR (2021) Children with attention-deficit/hyperactivity disorder spend more time in hyperconnected network states and less time in segregated network states as revealed by dynamic connectivity analysis. Neuroimage 229:117753. 10.1016/j.neuroimage.2021.117753 33454408 PMC7979530

[B93] Solomon M, Ozonoff SJ, Ursu S, Ravizza S, Cummings N, Ly S, Carter CS (2009) The neural substrates of cognitive control deficits in autism spectrum disorders. Neuropsychologia 47:2515–2526. 10.1016/j.neuropsychologia.2009.04.019 19410583 PMC2766616

[B94] Spadone S, Penna SD, Sestieri C, Betti V, Tosoni A, Perrucci MG, Romani GL, Corbetta M (2015) Dynamic reorganization of human resting-state networks during visuospatial attention. Proc Natl Acad Sci U S A 112:8112–8117. 10.1073/pnas.1415439112 26080395 PMC4491799

[B95] Sutoko S, Monden Y, Tokuda T, Ikeda T, Nagashima M, Kiguchi M, Maki A, Yamagata T, Dan I (2019) Distinct methylphenidate-evoked response measured using functional near-infrared spectroscopy during go/no-go task as a supporting differential diagnostic tool between attention-deficit/hyperactivity disorder and autism spectrum disorder comorbid children. Frontiers Hum Neurosci 13:7. 10.3389/fnhum.2019.00007 PMC6375904 30800062

[B96] Uddin LQ (2021) Brain mechanisms supporting flexible cognition and behavior in adolescents with autism spectrum disorder. Biol Psychiatry 89:172–183. 10.1016/j.biopsych.2020.05.010 32709415 PMC7677208

[B97] Uddin LQ, Supekar K, Lynch CJ, Cheng KM, Odriozola P, Barth ME, Phillips J, Feinstein C, Abrams DA, Menon V (2015) Brain state differentiation and behavioral inflexibility in autism. Cereb Cortex 25:4740–4747. 10.1093/cercor/bhu161 25073720 PMC4635916

[B98] Wakabayashi A, Baron-Cohen S, Wheelwright S, Tojo Y (2006) The autism-spectrum quotient (AQ) in Japan: a cross-cultural comparison. J Autism Dev Disord 36:263–270. 10.1007/s10803-005-0061-2 16586157

[B99] Watanabe T (2021) Causal roles of prefrontal cortex during spontaneous perceptual switching are determined by brain state dynamics. Elife 10:e69079. 10.7554/eLife.69079 34713803 PMC8631941

[B100] Watanabe T, Rees G (2017) Brain network dynamics in high-functioning individuals with autism. Nat Commun 8:16048. 10.1038/ncomms16048 28677689 PMC5504272

[B101] Watanabe T, Hirose S, Wada H, Imai Y, Machida T, Shirouzu I, Konishi S, Miyashita Y, Masuda N (2013) A pairwise maximum entropy model accurately describes resting-state human brain networks. Nat Commun 4:1370. 10.1038/ncomms2388 23340410 PMC3660654

[B102] Watanabe T, Masuda N, Megumi F, Kanai R, Rees G (2014) Energy landscape and dynamics of brain activity during human bistable perception. Nat Commun 5:4765. 10.1038/ncomms5765 25163855 PMC4174295

[B103] Watanabe T, Lawson RP, Walldén YSE, Rees G (2019a) A neuroanatomical substrate linking perceptual stability to cognitive rigidity in autism. J Neurosci 39:6540–6554. 10.1523/JNEUROSCI.2831-18.2019 31213484 PMC6697400

[B104] Watanabe T, Rees G, Masuda N (2019b) Atypical intrinsic neural timescale in autism. Elife 8:e42256. 10.7554/eLife.42256 30717827 PMC6363380

[B105] Wolff A, Berberian N, Golesorkhi M, Gomez-Pilar J, Zilio F, Northoff G (2022) Intrinsic neural timescales: temporal integration and segregation. Trends Cogn Sci 26:159–173. 10.1016/j.tics.2021.11.007 34991988

[B106] Yeo BTT, Krienen FM, Sepulcre J, Sabuncu MR, Lashkari D, Hollinshead M, Roffman JL, Smoller JW, Zöllei L, Polimeni JR, Fischl B, Liu H, Buckner RL (2011) The organization of the human cerebral cortex estimated by intrinsic functional connectivity. J Neurophysiol 106:1125–1165. 10.1152/jn.00338.2011 21653723 PMC3174820

[B107] Yerys BE, Bertollo JR, Pandey J, Guy L, Schultz RT (2019a) Attention-deficit/hyperactivity disorder symptoms are associated with lower adaptive behavior skills in children with autism. J Am Acad Child Adolesc Psychiatry 58:525–533.e3. 10.1016/j.jaac.2018.08.017 31029198

[B108] Yerys BE, Tunç B, Satterthwaite TD, Antezana L, Mosner MG, Bertollo JR, Guy L, Schultz RT, Herrington JD (2019b) Functional connectivity of frontoparietal and salience/ventral attention networks have independent associations with co-occurring attention-deficit/hyperactivity disorder symptoms in children with autism. Biol Psychiatry Cogn Neurosci Neuroimaging 4:343–351. 10.1016/j.bpsc.2018.12.012 30777604 PMC6456394

[B109] Yin W, Li T, Mucha PJ, Cohen JR, Zhu H, Zhu Z, Lin W (2022) Altered neural flexibility in children with attention-deficit/hyperactivity disorder. Mol Psychiatry 27:4673–4679. 10.1038/s41380-022-01706-4 35869272 PMC9734048

[B110] Young S, et al. (2020) Guidance for identification and treatment of individuals with attention deficit/hyperactivity disorder and autism spectrum disorder based upon expert consensus. Bmc Med 18:146. 10.1186/s12916-020-01585-y 32448170 PMC7247165

